# Evaluating the evidence for exponential quantum advantage in ground-state quantum chemistry

**DOI:** 10.1038/s41467-023-37587-6

**Published:** 2023-04-07

**Authors:** Seunghoon Lee, Joonho Lee, Huanchen Zhai, Yu Tong, Alexander M. Dalzell, Ashutosh Kumar, Phillip Helms, Johnnie Gray, Zhi-Hao Cui, Wenyuan Liu, Michael Kastoryano, Ryan Babbush, John Preskill, David R. Reichman, Earl T. Campbell, Edward F. Valeev, Lin Lin, Garnet Kin-Lic Chan

**Affiliations:** 1 Division of Chemistry and Chemical Engineering, California Institute of Technology, Pasadena, CA 91125 USA; 2 Department of Chemistry, Columbia University, New York, NY 10027 USA; 3 Department of Mathematics, University of California, Berkeley, CA 94720 USA; 4 AWS Center for Quantum Computing, Pasadena, CA 91125 USA; 5 Department of Chemistry, Virginia Tech, Blacksburg, VA 24061 USA; 6 Theoretical Division, Los Alamos National Laboratory, Los Alamos, NM 87545 USA; 7 Amazon Quantum Solutions Lab, Seattle, WA 98170 USA; 8 Google Quantum AI, 340 Main Street, Venice, CA 90291 USA; 9 Institute for Quantum Information and Matter, California Institute of Technology, Pasadena, CA 91125 USA; 10 Riverlane, Cambridge, UK; 11 Computational Research Division, Lawrence Berkeley National Laboratory, Berkeley, CA 94720 USA

**Keywords:** Quantum simulation, Quantum chemistry

## Abstract

Due to intense interest in the potential applications of quantum computing, it is critical to understand the basis for potential exponential quantum advantage in quantum chemistry. Here we gather the evidence for this case in the most common task in quantum chemistry, namely, ground-state energy estimation, for generic chemical problems where heuristic quantum state preparation might be assumed to be efficient. The availability of exponential quantum advantage then centers on whether features of the physical problem that enable efficient heuristic quantum state preparation also enable efficient solution by classical heuristics. Through numerical studies of quantum state preparation and empirical complexity analysis (including the error scaling) of classical heuristics, in both ab initio and model Hamiltonian settings, we conclude that evidence for such an exponential advantage across chemical space has yet to be found. While quantum computers may still prove useful for ground-state quantum chemistry through polynomial speedups, it may be prudent to assume exponential speedups are not generically available for this problem.

## Abstract (web summary)

The extent of problems in quantum chemistry for which quantum algorithms could provide a speedup is still unclear, as well as the kind of speedup one should expect. Here, the authors look at the problem of ground state energy estimation, and gather theoretical and numerical evidence for the fact that an exponential quantum advantage is unlikely for generic problems of interest.

## Introduction

The most common task in quantum chemistry is computing the ground electronic energy. The exponential quantum advantage (EQA) hypothesis for this task is that for a large set of relevant ("generic”) chemical problems, this may be completed exponentially more quickly (as a function of system size) on a quantum versus classical computer (for a representative sample of papers that refer to EQA, see ref. ^1^). Rigorously, computing the ground-state of even simple Hamiltonians can be exponentially hard on a quantum computer^2^. However, such Hamiltonians might not be relevant to generic chemistry. Thus, the specific exponential quantum advantage (EQA) hypothesis considered here, is that generic chemistry involves Hamiltonians which are polynomially easy for quantum algorithms (with respect to ground-state preparation) and simultaneously still exponentially hard classically, even using the best classical heuristics.

In this work, we examine the evidence for this EQA hypothesis, within the fault-tolerant quantum setting—the most advantageous setting for quantum computing. We do not attempt a rigorous proof (or disproof) as such proofs cannot be obtained, not least because what is “generic” chemistry is not precisely defined. Instead, we proceed using numerical experiments supported by theoretical analysis, and study whether heuristic quantum state preparation is exponentially easier than classical heuristic solution in typical problems; and whether the cost of classical heuristics in such problems, for a given error, scales exponentially with system size.

## Results

### Statement of the problem

We compute the ground-state eigenvalue *E* of the electronic Schrödinger operator (Hamiltonian) of a chemical system discretized with a basis set, and the problem size is the basis size *L*. We consider the case where increasing *L* corresponds to increasing physical system size (i.e., number of atoms) with basis size proportional to system size (other scenarios are discussed in Supplementary Note 2.1). The absolute ground-state energy *E* increases with *L*, and in physical problems we expect extensivity (i.e. $\mathop{\lim }\limits_{L\to \infty }E(L)\propto L$ for a chemically uniform system); in this limit, the energy density $\bar{E}=E/L$ may be the quantity of interest. Thus depending on the setting, the relevant error can be *ϵ* (error in *E*) or $\bar{\epsilon }$ (error in $\bar{E}$).

### Fault-tolerant algorithms for ground-state quantum chemistry

Fault-tolerant quantum algorithms are ones which employ deep circuits (e.g., depth is a function of 1/*ϵ*). The most famous one in quantum chemistry is quantum phase estimation (QPE)^3,4^. We focus on QPE for simplicity; qualitative features of the complexity remain similar in “post-QPE” algorithms^5^. Phase estimation approximately measures the energy with approximate projection onto an eigenstate. The cost has 3 components (i) preparing an initial state Φ, (ii) the phase estimation circuit, and (iii) the number of repetitions (to produce the ground-state Ψ_0_ rather than any eigenstate). The cost to obtain *E* to precision *ϵ* is

1 $$\,{{\mbox{poly}}}\,(1/S)[\,{{\mbox{poly}}}(L){{\mbox{poly}}}\,(1/\epsilon )+C]$$

where *C* corresponds to (i), poly(*L*)poly(1/*ϵ*) corresponds to (ii), and poly(1/*S*) ( = 1/*S*^2^ for QPE) with *S* = ∣〈Φ∣Ψ_0_〉∣ corresponds to (iii). We term *S* overlap and *S*^2^ weight.

Motivated by the poly(*L*) cost of (ii), and assuming an $\exp (L)$ cost for classical solution, it is often informally stated that QPE yields EQA for the ground-state quantum chemistry task^1^. However, the number of repetitions (poly(1/*S*)) may also depend on *L*: this stems from the quality of state preparation. The restriction to generic chemistry effectively means we assume that good state preparation is not exponentially hard due to unspecified additional structure. But such additional structure could also aid classical heuristics, and for EQA, the state preparation cost must be exponentially less than the classical solution cost.

### State preparation and EQA

As a first state preparation heuristic, we can prepare a state specified by an approximate classical ansatz (ansatz state preparation). (We assume once an ansatz solution is specified, it is easy to prepare on the quantum device.) Often, simple states, such as the Hartree-Fock or Kohn-Sham ground-state (single Slater determinants) are considered in ansatz state preparation, as they are hoped to have good overlap with Ψ_0_^6,7^; the poly(1/*S*) cost is then not further quantitatively considered. But while good overlap with such simple states can be observed in small molecules, EQA is an asymptotic statement, thus we should consider the limit of large *L*.

The orthogonality catastrophe^8,9^ has previously been discussed in the context of state preparation in the large *L* limit^10^. For a set of *O*(*L*) non-interacting subsystems, the global ground-state is the product of the subsystem ground-states, thus if the local overlap between the approximate classical ansatz and ground-state for each subsystem is ~ *s* < 1, then the global overlap is *s*^*O*(*L*)^ i.e., it decreases exponentially. This is sometimes viewed as an obstacle to ansatz state preparation, but in fact it does not rule it out; the issue is more subtle, because the above analysis assumes that both the ansatz and the actual ground-state have some product structure. But one need not consider a classical ansatz with (approximate) product structure; and, at least in principle, ground-state correlations could mean that the global overlap is not guaranteed to be well approximated by a product of local overlaps. Also, even if one uses a product-like ansatz to approximate a ground-state of near-product form, one can improve the local overlap as a function of *L*, such that the global overlap is 1/poly(*L*) or better.

The relevant consideration for EQA, however, is that if classical heuristics can efficiently prepare states with such good overlap for large *L* (for some systems), they may also efficiently obtain the ground-state energy to the desired precision.

As an alternative to ansatz state preparation, we can prepare the state adiabatically (adiabatic state preparation (ASP)). This means, we evolve slowly from the ground-state of a solvable initial Hamiltonian to that of the desired Hamiltonian^4,11–13^. This requires that the ground-state gap along the path be not too small; for paths where the smallest minimum gap ${\Delta }_{\min }\ge 1/\,{{\mbox{poly}}}\,(L)$ (which we will term “protected”), ASP plus QPE provides a polynomial cost quantum algorithm. Since a protected gap is not guaranteed using an arbitrary initial Hamiltonian and path, ASP is a heuristic quantum algorithm. An extreme problem that expresses the difficulty of finding a good path is unstructured search, where ${\Delta }_{\min }$ acquires a strong dependence on the ground-state ϒ_0_ of the initial Hamiltonian, ${\Delta }_{\min } \sim|\langle {\Upsilon }_{0}|{\Psi }_{0}\rangle|$^14^, yielding exponential cost when using adiabatic algorithms.

The above raises several issues. First, in correlated quantum systems with competing ground-states, different phases could be separated by first-order phase transitions (where the gap is not protected) requiring ASP to start in the correct phase. Assuming one uses classical heuristics to prepare such a starting point and choice of path, one encounters similar questions to those raised in the discussion of ansatz state preparation. Second, one might ask how common the above situation is in generic chemistry, i.e., whether interesting chemical problems allow for initial Hamiltonians and paths with a protected gap to be trivially found.

### The power of classical heuristics

"Exact” classical methods for ground-state determination, such as exact diagonalization, are exponentially expensive on a classical computer (see Supplementary Note 2.3). Thus the typical methods used in quantum chemistry are classical heuristics, which come in a wide variety for different problems (see Supplementary Note 2.2). The critical questions for EQA are (i) do these heuristics require $\exp (L)$ cost for given *ϵ* or $\bar{\epsilon }$ in their application domain, (ii) does the patchwork of heuristics cover chemical space, and (iii) if there are gaps in coverage in practice, do we require classical methods of $\exp (L)$ cost to cover them?

EQA assumes exponential-scaling cost of classical heuristic algorithms for given *ϵ* (or $\bar{\epsilon }$) across generic problems. We will examine this assumption in our numerical experiments. However, as actually employed in calculations, classical heuristics are often executed with poly(*L*) cost without necessarily achieving a specific accuracy, complicating the comparison with rigorous quantum algorithms. In particular, the error dependence can impact the EQA comparison, for example, a $\,{{\mbox{poly}}}\,(L)\exp ({\bar{\epsilon }}^{-1})$ classical algorithm implies $\exp (L)$ cost for given *ϵ*. Thus we will also examine the empirical precision dependence of classical heuristics with respect to *ϵ* or $\bar{\epsilon }$.

### Analysis of state preparation in Fe-S clusters of nitrogenase

Iron-sulfur (Fe-S) clusters are amongst the most common bioinorganic motifs in Nature^15^, and the Fe-S clusters of nitrogenase have become a poster child problem for quantum chemistry on quantum devices^16,17^. In the current context, they provide a concrete setting to assess the EQA hypothesis, in particular, the behavior of quantum state preparation strategies.

**Fig. 1:**
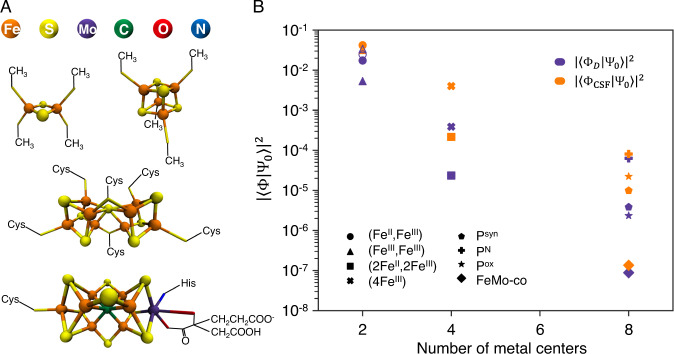
Ansatz state preparation and ansatz weights for model Fe-S clusters. **A** Structural models of [2Fe-2S], [4Fe-4S], P-cluster, and FeMo-co. **B** Weight of two different types of ansatz state: largest weight determinant (Φ_*D*_) (purple) and largest weight configuration state function (Φ_CSF_) (orange) as a function of the number of metal centers in each cluster (using split-localized orbitals). P^N^, P^syn^, P^ox^ here refer to different oxidation states of the metal ions in the P-cluster. Both types of ansatz state show an exponential decrease in weight with the number of metal centers. For the [2Fe-2S] clusters, we also show results for the largest weight determinant using natural orbitals (empty symbols).

Specifically, we consider iron-sulfur clusters containing 2, 4, 8 transition metal atoms (the latter includes the famous FeMo-cofactor) in Fig. 1. The 2, 4 metal clusters will be referred to as [2Fe-2S], [4Fe-4S] clusters, while the 8 metal clusters include the P-cluster (8Fe) and the FeMo-cofactor (7Fe, 1Mo). We note that the P-cluster and FeMo-cofactor are the largest Fe-S clusters found in Nature. We represent the electronic structure in active spaces with Fe 3d/S 3p character constructed from Kohn–Sham orbitals. Within the occupation number to qubit mapping, this corresponds to up to 40 qubits ([2Fe-2S]), up to 72 qubits ([4Fe-4S]), and up to 154 qubits (P-cluster and FeMo-co) (see Supplementary Note 4.1). For [2Fe-2S], exact solutions can be obtained using exact full configuration interaction (FCI). For all clusters, we obtain a range of approximate solutions using the quantum chemistry density matrix renormalization group (DMRG)^18–22^ with a matrix product state (MPS) bond dimension *D*; increasing *D* improves the approximation, allowing extrapolation to the exact result (see Supplementary Note 4.3). Note that the classical calculations in this section are of interest mainly to provide data to understand quantum state preparation.

We first examine the nature of the ground-state Ψ_0_ and the cost of ansatz state preparation. For this, we compute the weight of a Slater determinant *S*^2^ = ∣〈Φ_*D*_∣Ψ_0_〉∣^2^, shown in Fig. 1B. Φ_*D*_ is parametrized by its orbitals {*ϕ*}, and we choose a priori, or optimize, {*ϕ*} to maximize this weight (for a best-case scenario that uses information from the solution Ψ_0_, see Supplementary Note 4.4). The weights decrease exponentially over a small number of metal centers, and are already very small in FeMo-co (~10^−7^). The number of QPE repetitions is poly(1/*S*), yielding a large prefactor even for an “optimized” Slater determinant.

We next prepare more complex states with better overlap. We use a single configuration state function (a linear combination of Slater determinants that is an eigenfunction of total spin^23^). The weights improve but still show exponential decay to small values.

These results indicate that the magnitude of the ansatz overlap can become a relevant concern even in systems of modest size when using ansatz state preparation, thus we should consider improved state preparation. Assuming the ground-state is of approximately product form, we can obtain some rough insight into improved global ansatz state preparation (e.g., for eight metal clusters) from the behavior of improving the state locally (i.e., for the two metal clusters); we require poly(1/*I*) cost for the [2Fe-2S] fragment (*I* is the infidelity 1 − *S*^2^) for efficient global state preparation. In Supplementary Note 3.1 we show that this cost is indeed poly(1/*I*), but also that the energy error is poly(*I*). Thus under these assumptions, improving the local overlap sufficiently also implies efficient classical solution for the global energy. For any finite system, it may be possible to engineer a practical quantum advantage for some target precision from a sufficiently good ansatz overlap and a favorable ratio of classical and quantum costs. But the problem of finding a classical heuristic that efficiently yields 1/poly(*L*) overlap but which cannot also efficiently reach the target precision remains.

We next compute the ASP cost for a simplified *n*_act_ = 12 active space (24 qubit) [2Fe-2S] model (see Supplementary Note 5.1). The path is a heuristic input, and we use one which linearly interpolates the Hamiltonian *H*(*s*) (with ground-state ϒ_0_(*s*)) between an initial Hamiltonian (*s* = 0, with ground-state ϒ_0_(0)) and the true Hamiltonian (*s* = 1, with ground-state ϒ_0_(1) ≡ Ψ_0_); the path preserves spin symmetry. We consider two families of *H*(0); a set of mean-field Hamiltonians (with different Slater determinant ground-states) and a set of interacting Hamiltonians (these retain interactions among *q* active spin-orbitals (qubits), definitions in Supplementary Note 5.2).

**Fig. 2:**
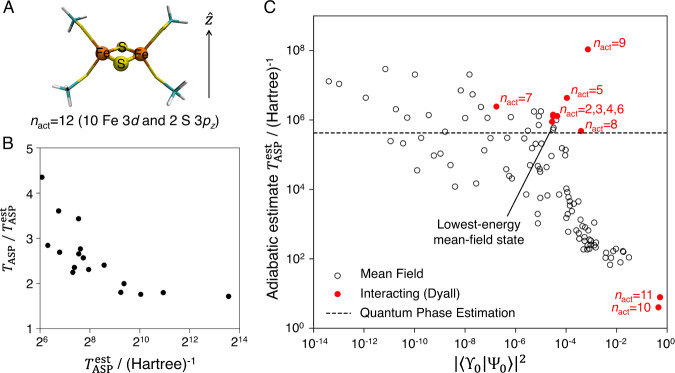
Adiabatic state preparation for a model [2Fe-2S] cluster. **A** Structure and simplified active space model of [2Fe-2S] cluster. **B** ASP time and the adiabatic estimate. We see that the ratio ${T}_{{{{{{{{\rm{ASP}}}}}}}}}/{T}_{{{{{{{{\rm{ASP}}}}}}}}}^{{{{{{{{\rm{est}}}}}}}}}$ is *O*(1). **C** Adiabatic estimates (${T}_{{{{{{{{\rm{ASP}}}}}}}}}^{{{{{{{{\rm{est}}}}}}}}}$) for two families of initial Hamiltonians against the weight of the initial ground state (ϒ_0_) in the final ground state (Ψ_0_) (∣〈ϒ_0_∣Ψ_0_〉∣^2^), showing an inverse dependence on the initial weight. The mean-field Hamiltonians are constructed to have different Slater determinants as their ground-state, while the interacting Hamiltonians contain the full electron interaction amongst *n*_act_ orbitals. Additional discussion in Supplementary Notes 3.3 and 5.

Tight bounds on the ASP time (*T*_ASP_) are difficult to obtain (see Supplementary Note 2.5). However, we have verified that the adiabatic estimate ${T}_{{{{{{{{\rm{ASP}}}}}}}}}^{{{{{{{{\rm{est}}}}}}}}} \sim \mathop{\max }\limits_{s}\tau (s)$, with *τ*(*s*) = ∣〈ϒ_0_(*s*)∣*dH*(*s*)/*ds*∣ϒ_1_(*s*)〉∣/Δ^2^(*s*) with Δ(*s*) the ground-state gap and ϒ_1_(*s*) the first excited state of *H*(*s*), is a reasonable estimate for the desired preparation fidelity (here assumed 75% final weight) by carrying out time-dependent simulations of ASP for simple instances to compute ${T}_{{{{{{{{\rm{ASP}}}}}}}}}/{T}_{{{{{{{{\rm{ASP}}}}}}}}}^{{{{{{{{\rm{est}}}}}}}}}$ (Fig. 2B); for a range of examples, this ratio is *O*(1). Thus we use ${T}_{{{{{{{{\rm{ASP}}}}}}}}}^{{{{{{{{\rm{est}}}}}}}}}$ as the ASP time below.

Figure 2C shows *T*_ASP_ across the sets of *H*(0); it varies over eight orders of magnitude depending on the choice of *H*(0). We see a trend $1/(\mathop{\min }\limits_{s}\Delta (s)) \sim \,{{\mbox{poly}}}\,(1/|\langle {\Upsilon }_{0}|{\Psi }_{0}\rangle|)$ and thus *T*_ASP_ ~ poly(1/∣〈ϒ_0_∣Ψ_0_〉∣) reminiscent of unstructured search. The practical consequence is that an a priori good choice of initial Hamiltonian is non-trivial; the mean-field Hamiltonian with the lowest ground-state energy gives a large *T*_ASP_ > *T*_QPE_ (an estimate of the total coherent QPE evolution time for 90% confidence, *ϵ* = 10^−3^*E*_*h*_, see analysis in Supplementary Note 3.5), while out of the interacting *H*(0)’s, we need to include almost all the interactions when diagonalizing *H*(0) for the initial state (20 out of 24 qubits) before *T*_ASP_ < *T*_QPE_. Although these results are for the smallest FeS cluster, the dependence of *T*_ASP_ on *S* is problematic for EQA should it scale to larger interesting problems, and it illustrates the importance of heuristics to find a good initial starting point for ASP in relevant chemical problems. As discussed above, if classical heuristics are used for this task, this raises the question of whether they are exponentially advantageous over the classical heuristics for solution.

### Empirical complexity analysis of classical heuristics

The Fe-S cluster simulations raise questions as to whether high quality quantum state preparation can be assumed to be exponentially easier than classical heuristic solution. We now consider if classical heuristics in fact display $\exp (L)$ cost for fixed *ϵ* or $\bar{\epsilon }$, as is required to establish EQA. We do so by considering examples that arguably represent much of chemical space, which are evidence of classical heuristics scaling to large problems and high accuracy at polynomial cost for fixed $\bar{\epsilon }$. (If the error scaling is $\,{{\mbox{poly}}}\,(1/\bar{\epsilon })$ independent of *L*, this further implies poly(*L*) cost overhead to achieve fixed *ϵ*). We note that the calculations below represent only a small slice of relevant evidence from classical calculations; related calculations can be found in the literature, although our focus here is on characterizing the calculations e.g. with respect to cost and precision in a way useful for understanding EQA. Some further discussion of these systems and other calculations relevant to EQA is in Supplementary Note 3.6.

**Fig. 3:**
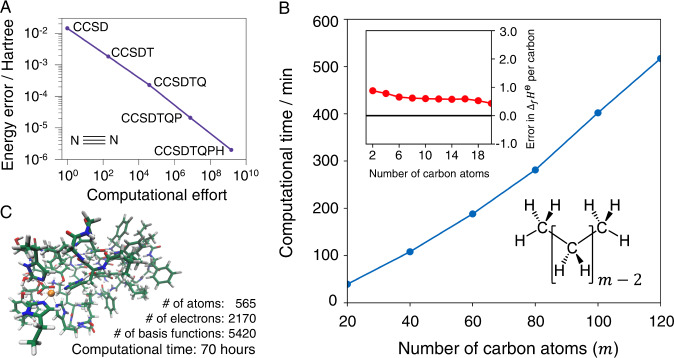
Computational complexity of classical heuristics for molecular systems. **A** Energy error of a nitrogen molecule (equilibrium geometry) as a function of the level of CC approximation, against a computational time metric. Data taken from ref. ^37^, time metric defined in Supplementary Note 3.7. The observed precision cost is like poly(1/*ϵ*). **B** Cost of a state-of-the-art reduced-scaling coupled-cluster (CCSD(T)) implementation scales nearly-linearly with the system size in gapped systems, as demonstrated here for *n*-alkanes (C_*m*_H_2*m*+2_) with *m* = [20…120]. Size-extensivity of the coupled-cluster ansatz ensures constant error per system subunit, as illustrated in the subfigure for the error of explicitly-correlated reduced-scaling CCSD(T)^34^ (see Supplementary Note 6.1 for details) with respect to the available experimental gas-phase enthalpy of formation in the standard state for *n*-alkanes with *m* = [2…20]. **C** Reduced-scaling CCSD(T) implementations can be routinely applied to systems with thousands of electrons on a few computer cores, as demonstrated here for a small fragment of photosystem II^38^.

For “single-reference” chemical problems (see Supplementary Note 6) coupled-cluster (CC) wavefunctions are often described as the gold-standard. The heuristic assumes that correlations of many excitations can be factorized into clusters of fewer excitations; if the maximal cluster excitation level is independent of *L*, the cost is poly(*L*) (assuming a non-exponential number of iterations for solution) without guaranteed error. To establish the error dependence, Fig. 3A shows the empirical convergence of error as a function of cost, consistent with poly(1/*ϵ*) scaling, for a small molecule (N_2_). By the extensivity of the coupled-cluster wavefunction, this translates to $\,{{\mbox{poly}}}(L){{\mbox{poly}}}\,(1/\bar{\epsilon })$ cost for a gas of non-interacting N_2_ molecules, and thus poly(*L*)poly(1/*ϵ*) given the error convergence above. We can take this as a conjectured complexity of coupled cluster in single-reference problems. To practically test this scaling form on larger systems, we introduce another heuristic. CC methods can be formulated to exploit locality, a commonly observed and widely conjectured feature of physical ground-states (including gapless systems, see Supplementary Note 2.4). This yields the local CC heuristic that has cost linear in *L* in gapped systems^24,25^. Figure 3B illustrates the application of local CC to *n*-alkanes, a set of organic molecules, with the associated computational timing. This suggests the cost is *O*(*L*), while the computed enthalpy of formation per carbon achieves constant error versus experimental data, reflecting constant $\bar{\epsilon }$ as a function of *L*, consistent with the conjectured complexity. Many biomolecules are single-reference problems, allowing local coupled-cluster methods to be applied to protein-fragment-scale simulations (Fig. 3C).

Strongly correlated materials (e.g., bulk analogs of the Fe-S clusters) remain challenging to treat with ab initio quantum chemistry (although there has been considerable progress in recent years^26,27^). To obtain insight into the computational complexity, it is more practical to study simpler models of correlated materials (e.g., the Heisenberg and Hubbard models, often used to study quantum magnets and high temperature superconductors^28,29^). Many methods can now access large parts of these model phase diagrams to reasonable accuracy without $\exp (L)$ cost. The use of locality is common to several heuristics for strongly correlated problems; tensor networks are an example of such a class of heuristics and we examine illustrative applications below. (Note that this is not an exhaustive study of tensor networks, nor of other heuristics (such as quantum embedding); for additional discussion see Supplementary Note 3.6).

**Fig. 4:**
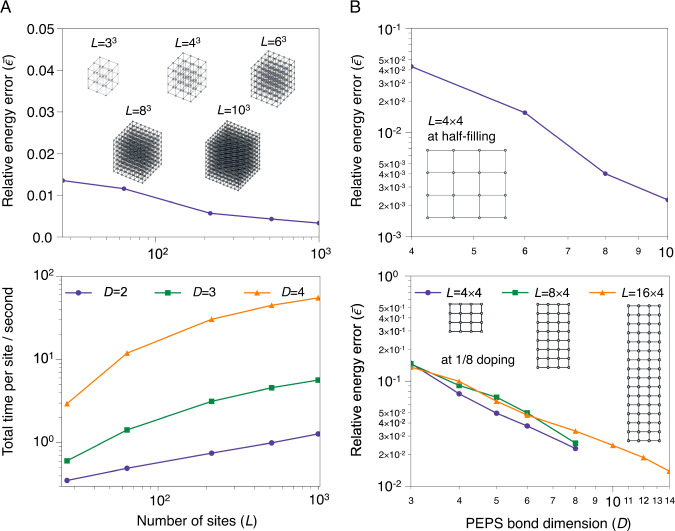
Computational complexity of classical heuristics for models of strongly correlated material systems. **A** Relative energy error of a tensor network (PEPS) with respect to system sizes 3^3^ to 10^3^ for the 3D Heisenberg cube model with a bond dimension (*D*) of 4. In bottom panel: total computational time in seconds, divided by number of sites, as a function of system size, demonstrating poly(*L*) (close to linear) computational effort. **B** Energy convergence of PEPS with respect to the bond dimension for 2D Hubbard models at half filling (4 × 4 lattice, in top panel) and the challenging 1/8 doping point (4 × 4, 8 × 4 and 16 × 4 lattice, in bottom panel). The plots are consistent with $1/\bar{\epsilon } \sim \,{{\mbox{poly}}}\,(D)$ with a weak dependence on *L*.

Figure 4 shows results from a tensor network ansatz^30^, where the expressiveness of the ansatz is controlled by the bond dimension *D*. The contraction here is explicitly performed with poly(*D*) (typically a high polynomial) cost, thus for given *D* (assuming the number of iterations is not exponential in *D* or *L* (see Supplementary Note 7)), the algorithm cost is poly(*L*)poly(*D*), without guaranteed error. $\bar{\epsilon }$ and computational cost are shown as a function of *L* in the 3D cubic Heisenberg model, and $\bar{\epsilon }$ as a function of *D* and *L* in the 2D Hubbard model. (Note: these examples were chosen for ease of generating exact data, rather than representing the limits of classical methods in size, accuracy, or complexity of physics; see Supplementary Note 3.6 for other examples). Figure 4A shows that the cost is close to *O*(*L*) in the 3D Heisenberg model for up to 1000 sites, while achieving close to constant $\bar{\epsilon }$. Less data is available for the error scaling as accessible *D* remain small; in particular it is currently too expensive to reach large enough *D* to meaningfully study the $\bar{\epsilon }$ scaling in 3D. However in the 2D Hubbard model (Fig. 4B) we see $\bar{\epsilon } \sim 1/\,{{\mbox{poly}}}\,(D)$ (or slightly better) across a range of studied *D*, (with a weak dependence on *L*) even at the challenging 1/8 doped point of the model. Assuming this error form holds asymptotically, the observed empirical cost is $\,{{\mbox{poly}}}(L){{\mbox{poly}}}(D){{\mbox{poly}}}\,(1/\bar{\epsilon })$, which corresponds to poly(*L*)poly(*D*)poly(1/*ϵ*) for the assumed error scaling, and we can conjecture that this holds also in 3D.

Although the Hubbard and Heisenberg models are believed to contain the basic physics of many strongly correlated materials, moving from such simplified models to more detailed quantum chemistry models (i.e., ab initio Hamiltonians) will certainly increase complexity. But establishing EQA requires evidence that adding the polynomial number of terms in the Hamiltonian causes the classical heuristic to fail or become exponentially expensive. The history of development of classical heuristics does not support this, as methods originally developed on simpler models routinely graduate to ab initio simulations. For example, the coupled-cluster methods described above were first developed for use in model simulations, as were simpler tensor networks such as the density matrix renormalization group and tree tensor networks now used in ab initio calculations^18,31,32^; Supplementary Note 3.6 provides more discussion of this point as well as shows the performance of a quantum embedding heuristic for the 2D and 3D hydrogen lattices, ab initio analogs of the 2D Hubbard and 3D Heisenberg systems in Fig. 4. Further examples in the literature consider the application of many different classical heuristics to ab initio or model chemical ground-states of complex systems including strongly correlated materials^22,25–27,29,33–36^. Although the computational complexity is not formally analyzed, the success of such studies of large and complex problems is compatible with the view that the ground-state quantum chemistry problem is often soluble with classical heuristics, to an energy density error $\bar{\epsilon }$ relevant to physical problems, with poly(*L*) cost. Thus, while there are many chemistry problems that cannot currently be addressed by classical methods and further study can be expected, the barrier to solution may be of polynomial (even if impractically large) rather than exponential cost.

## Discussion

We have examined the case for the exponential quantum advantage (EQA) hypothesis for the central task of ground-state determination in quantum chemistry. The specific version of EQA that we examined required quantum state preparation to be exponentially easy compared to classical heuristics, and for classical heuristics to be exponentially hard. Our numerical simulations highlight that heuristics are necessary to achieve efficient quantum ground-state preparation. At the same time, we do not find evidence for the exponential scaling of classical heuristics in a set of relevant problems. The latter suggests that quantum state preparation can be made efficient for the same problems. However, as EQA is based on the ratio of costs, this does not lead to EQA.

Numerical calculations are neither mathematical proof of asymptotics with respect to size and error, nor can we exclude EQA in specific problems. However, our results suggest that without new and fundamental insights, there may be a lack of generic EQA in this task. Identifying a relevant quantum chemical system with strong evidence of EQA remains an open question.

We neither consider tasks other than ground-state determination, nor do we rule out polynomial speedups. Depending on the precise form, polynomial quantum speedups could be associated with useful quantum advantage, as even a polynomial classical algorithm does not mean that solutions can be obtained in a practical time. Both aspects may prove important in the further development of quantum algorithms in quantum chemistry. For further discussion, we refer to the FAQ (see Supplementary Note 1).

## Supplementary information

Supplementary Information

Peer Review File

## Data Availability

The FCI/DMRG data for state preparation in Fe-S clusters of nitrogenase are available in Supplementary Notes 3.1, 3.2 and 4.1–4.6. The ASP data are available in Supplementary Notes 3.3, 5.1, and 5.2. The CC data are available in Supplementary Notes 6.1 and 6.2 The PEPS DMRG/VMC data are available in Supplementary Notes 7.1–7.3.

## Code availability

Source codes are available from the authors on request.
