# Supplementary material for: Evaluating the evidence for exponential quantum advantage in ground-state quantum chemistry
Source: Nat Commun. 2023 Apr 7;14:1952. doi: 10.1038/s41467-023-37587-6 (PMC10082187; doi:10.1038/s41467-023-37587-6)
Supplement: Supplementary file 1 — Supplementary Information [file 41467_2023_37587_MOESM1_ESM.pdf]

Supplementary Information for Evaluating the evidence for exponential quantum advantage in ground-state quantum chemistry

Seunghoon Lee[1], Joonho Lee[2], Huanchen Zhai[1], Yu Tong[3], Alexander M. Dalzell[4], Ashutosh Kumar[5,6], Phillip Helms[1], Johnnie Gray[1], Zhi-Hao Cui[1], Wenyuan Liu[1], Michael Kastoryano[4,7], Ryan Babbush[8], John Preskill[4,9], David R. Reichman[2], Earl T. Campbell[10], Edward F. Valeev[5], Lin Lin[3,11], and Garnet Kin-Lic Chan[1,*]

[1]Division of Chemistry and Chemical Engineering, California Institute of Technology, Pasadena, California 91125, USA
[2]Department of Chemistry, Columbia University, New York, New York 10027, USA
[3]Department of Mathematics, University of California, Berkeley, California 94720, USA
[4]AWS Center for Quantum Computing, Pasadena, California 91125, USA
[5]Department of Chemistry, Virginia Tech, Blacksburg, Virginia 24061, USA
[6]Theoretical Division, Los Alamos National Laboratory, Los Alamos, NM 87545, USA
[7]Amazon Quantum Solutions Lab, Seattle, Washington 98170, USA
[8]Google Quantum AI, 340 Main Street, Venice, California 90291, USA
[9]Institute for Quantum Information and Matter, California Institute of Technology, Pasadena, California 91125, USA
[10]Riverlane, Cambridge, UK
[11]Computational Research Division, Lawrence Berkeley National Laboratory, Berkeley, California 94720, USA
[*]Corresponding author: gkc1000@gmail.com

Contents

1 Frequently asked questions (FAQ) 4

2 Theoretical background 5

2.1 Basis size scaling . . . 5

2.2 Overview of classical heuristics . . . 6

2.3 Complexity of rigorous quantum and classical methods for quantum chemistry under the assumption of efficient state preparation . . . 7

2.4 Locality and area laws . . . 9

2.5 Sufficient conditions for the validity of the adiabatic approximation . . . 11

3 Supporting discussion and data for numerical experiments 12

3.1 Additional ansatz state preparation data . . . 12

3.2 Scaling of DMRG bond dimension in the Fe-S clusters . . . 13

3.3 Additional discussion of adiabatic state preparation data . . . 14

3.4 Time-dependent simulations of adiabatic state preparation . . . 16

3.5 Evolution time for quantum phase estimation . . . 18

3.6 Overview of systems chosen for scaling of classical heuristics . . . 19

3.7 Error scaling and cost of local coupled cluster theory . . . 22

4 Technical details: electronic ground-state calculations for the Fe-S clusters 23

4.1 Active space models . . . 23

4.2 Initial guesses for DMRG calculations . . . 24

4.3 Extrapolations for the total DMRG energies . . . 26

4.4 Extracting the largest weight determinant and configuration state function . . . 28

4.5 Computational time metric 29

4.6 Additional convergence data for the [2Fe-2S] clusters 31

5 Technical details: Adiabatic state preparation 32

5.1 CAS model for adiabatic state preparation 32

5.2 Initial Hamiltonian for the adiabatic state preparation 33

6 Local coupled cluster calculations 34

6.1 The coupled-cluster-based protocol for the enthalpy of formation of n-alkanes . 35

6.2 Computational details of the local coupled-cluster computations 37

7 Tensor network calculations 38

7.1 PEPS-DMRG Hubbard calculations 38

7.2 PEPS-VMC Hubbard calculations 39

7.3 3D Heisenberg model PEPS reference calculations 42

Supplementary Note 1 Frequently asked questions (FAQ)

1. *Are there any published papers which suggest exponential quantum advantage for ground-state quantum chemistry?* Statements of this kind can be found in various settings, and range from direct statements of EQA for ground-state problems, to more implicit statements where the expectation of EQA could reasonably be inferred by the reader. Some representative papers and further discussion can be found in Ref. [1].

2. *Does this mean that quantum computers are not useful for generic ground-state quantum chemistry?* Even if EQA is not found in the ground-state problem, quantum computers may still be useful for this task, since polynomial advantage (or even large constant factor advantage) can be very useful. In such cases, the details (e.g. degree of polynomial, size of constant) for both the classical heuristic and quantum algorithm are important. It is difficult to tie generic statements about advantage to other characteristics, such as strength of correlation; for example, in more strongly correlated systems, state preparation must be more carefully considered due to possible competing phases. However, in terms of assessing the suitability of ground-state quantum chemistry as an early target application for quantum computers, one should compare the degree of available polynomial advantage in quantum chemistry to that in other applications.

3. *We do not have answers to a certain problem by classical heuristics, and exact classical solution is exponentially expensive, doesn't this mean that there should be EQA?* As our work indicates, this is a subtle question. First, one can definitely construct artificial ground-state problems which, for certain precision requirements, can be solved efficiently quantumly but not classically. See e.g. Supplementary Note 2.3 as well as Refs. [2, 3]. The open question there is whether such problems are related to the generic molecules and materials studied in quantum chemistry. Second, there are relevant chemical prob-

lems which are too large to treat with current classical heuristics with the desired precision. However, for EQA, one must establish that e.g. to reach the desired precision, the classical heuristic requires exponential effort as a function of system size, and also that quantum algorithms are capable of reaching the desired precision without exponential effort.

4. *What about potential improvements to quantum algorithms?* We cannot anticipate future improvements to quantum algorithms, for example, in the area of quantum heuristics. However, to change the situation regarding EQA it is critical for an improvement to change the ratio of the quantum to classical costs. For example, when introducing locality into a quantum heuristic, we should understand whether classical heuristics that also use locality are efficient for the same problems.

5. *What about other classes of problems such as quantum dynamics for chemical systems?* We cannot conclude anything about other simulation tasks based on this work. However, we note that other tasks, such as the simulation of chemical dynamics, may also be amenable to heuristics because of the particular chemical setting and question of interest; for example, dynamics of heavier atoms can often be treated classically, or certain phenomena may take place with strong dissipation. Thus heuristics should always be carefully considered in claims of EQA and other types of quantum advantage.

Supplementary Note 2 Theoretical background

2.1 Basis size scaling

In the main text, we described scaling with L where L is the physical system size. However, we can also consider increasing the basis size L while keeping the physical system size fixed. One can view this as increasing the spatial resolution with which one is representing the sys-

tem. This type of scaling exhibits very different characteristics to scaling the physical system size. For example, we expect the energy to (smoothly) converge to the basis set limit result, a finite number (i.e. $\lim_{L\to\infty} E(L) \to E(\infty)$). For a fixed error ϵ (with respect to $E(\infty)$), the computational cost should become independent of L for sufficiently large L, as one can simply neglect any additional basis functions. Similarly, past a certain basis size, we do not expect the ground-state to change much, thus the state preparation cost can also become independent of L for sufficiently large L. For the chemical Hamiltonian, the asymptotic rate of convergence of the exact $E(L)$ is known to be controlled by the singularity in the electron-electron interaction at zero-separation which creates a cusp in the wavefunction along the electron-electron coordinate, see e.g. Ref. [4].

2.2 Overview of classical heuristics

There are many types of classical heuristics for quantum chemistry and quantum many-body problems. They are too numerous to describe here, we only give a short overview with some references. A non-exhaustive list includes empirically parameterized methods, such as density functional methods [5] (which are generally of modest cost but which are not systematically improvable) to non-parametrized many-body methods, which usually have some computational parameter which may be tuned such that $\epsilon \to 0$. Out of the latter, there are methods based on perturbing around mean-field wavefunctions (this includes standard coupled cluster theory) [6], various flavours of quantum Monte Carlo methods [7, 8], systematically improvable variational wavefunctions, such as configuration interaction wavefunctions [6], tensor network states [9, 10], and neural network wavefunctions [11, 12], and reduced density matrix approaches based on N-representability constraints [13]. Each method makes tradeoffs to avoid the exponential scaling of exact methods, and the quality of these tradeoffs defines different optimal domains of application. Recent years have seen benchmark studies comparing a wide variety of different

heuristic methods, see e.g. Refs. [14, 15, 16].

2.3 Complexity of rigorous quantum and classical methods for quantum chemistry under the assumption of efficient state preparation

The main text highlights the importance of both quantum and classical heuristics in establishing EQA, and that quantum advantage in practice is related to the relative cost of (heuristic) quantum state preparation versus classical heuristics for the ground-state problem.

If, however, we separate the problem of state preparation from that of ground-state determination (i.e. we assume that a good initial state is available, which may or may not be efficiently preparable by classical or quantum means) then it is possible to establish conditions which demonstrate a clear separation between rigorous quantum and classical algorithms, i.e. algorithms with error guarantees.

For example, in estimating the ground state energy, QPE and other related quantum algorithms have a rigorous precision guarantee under the assumption of having an initial guess with good overlap. If we require classical methods to have the same rigorous precision guarantee, under the same assumption, then we can construct problems where quantum algorithms can have a very significant advantage. Here we consider the setting where we have a good initial guess $|\Phi_0\rangle$ for the ground state $|\Psi_0\rangle$ with overlap $S = |\langle\Phi_0|\Psi_0\rangle|$ that is lower bounded by $1/\operatorname{poly}(L)$, where L is the system size.

On a classical computer, brute-force diagonalization to compute the ground-state energy will result in $\exp(L)$ runtime and memory requirements. However, there are more clever ways to obtain the ground state energy. One can compute quantities of the form $\|f(H)|\Phi_0\rangle\|$ for polynomials f, and this enables us to classically implement the binary search procedure described in Ref. [17]. With this method, let us consider a setting where the Hamiltonian, relative to a suitable computationally accessible basis, has $\operatorname{poly}(L)$ non-zero elements in each row and column,

while $|\Phi_0\rangle$ contains $\mathcal{O}(1)$ non-zero elements relative to the same basis. The number of matrix-vector multiplications needed is $\mathrm{polylog}(\|H\|\epsilon^{-1})$, corresponding to the sum of the degrees of the polynomials f we need to implement. Each time matrix-vector multiplication is performed, the number of non-zero elements of the vector grows by a factor of $\mathrm{poly}(L)$ which corresponds to the sparsity of the Hamiltonian. The total runtime will in the end be $\mathrm{poly}(L)^{\mathrm{polylog}(\|H\|\epsilon^{-1})}$, where $\|H\| = \mathrm{poly}(L)$. In a similar setting, QPE and other related quantum algorithms will have a $\mathrm{poly}(L)\epsilon^{-1}$ runtime. Therefore at least for certain Hamiltonians and guarantees about the initial state, there can be a significant advantage for a quantum algorithm versus a classical algorithm with the same rigorous error guarantee.

The appearance of $\mathrm{polylog}(||H||\epsilon^{-1})$ in the above exponent suggests that, assuming the availability of an initial state with good overlap, there is hope of having a polynomial time classical algorithm with guaranteed performance for the ground state energy only when constant *relative* precision $\|H\|\epsilon^{-1}$ (which, if $||H|| = \mathcal{O}(L)$ means constant precision in $\bar{\epsilon}$) is required. Indeed, such a result was recently obtained (Theorem 1 in Ref. [2]) which established that estimating the ground state energy of a local Hamiltonian to constant relative precision can be achieved in polynomial time on a classical computer, assuming sampling access to an initial guess of the ground state. Note that we did not require computing a description of the ground state to any precision. If the ground state is required, then using methods such as the Lanczos iteration, a relative spectral gap dependence (relative to $||H||$) will need to be introduced, making the runtime scale like $\mathrm{poly}(L)^{\mathrm{poly}(\|H\|)\,\mathrm{polylog}(\|H\|\epsilon^{-1})}$. For this task, the runtime of QPE and other related quantum algorithms will still be $\mathrm{poly}(L)\epsilon^{-1}$.

Ref. [2] established another separation between rigorous quantum and classical algorithms. Assuming access to a good initial guess of the ground state, Theorem 2 of Ref. [2] showed that estimating the ground-state energy of certain Hamiltonians to inverse-polynomial absolute (and relative) precision is BQP-hard. The proof proceeds by using a circuit-to-Hamiltonian construc-

tion [18] to encode a polynomial-time BQP computation into a local Hamiltonian, where the ground state energy is related to whether the answer to the BQP problem is yes or no; because the relevant gap of the problem vanishes inversely polynomially with system size, the ability to estimate the energy to inverse polynomial precision using a classical algorithm would imply $\mathsf{BQP} = \mathsf{BPP}$, which is generally regarded as unlikely. Certain aspects of this construction may not necessarily be common in physical problems. For example, although there has been considerable effort to make the Hamiltonians involved more physically reasonable (for example, it has been shown that they can be composed of nearest-neighbor interactions on a 1D (high-local dimension) spin chain [19], and these interactions can even be translationally invariant [20]) it remains unclear whether they arise naturally in physical settings. In general, the relevant ground states are critical systems whose spectral gaps close inverse-polynomially, and moreover, by construction, their ground states are highly entangled (as they encode the states encountered by an arbitrary quantum computation). This contrasts with generic systems in quantum chemistry which might be expected to obey an area law (see Supplementary Note 2.4). This aspect, along with the need for inverse-polynomial absolute precision on the energy estimate [21], complicates any concrete conclusions that can be drawn from Ref. [2] about the prospect of rigorous EQA in quantum chemistry, even under the assumption of efficient state preparation.

2.4 Locality and area laws

Locality and entanglement area laws. The extensive energy as $L \to \infty$ is a result of the long-range decay (locality) of interactions between electrons and the charge neutrality of the system. This type of locality might be termed Hamiltonian locality (and is closely related to the more formal idea of geometric locality in lattice systems).

A related statement of locality for quantum states is the area "law" of entanglement for ground-states (strictly a conjecture in the general setting) which states the entanglement entropy

for a bipartition is proportional to the length of the boundary, with at most logarithmic violations for critical states (e.g. metallic states). Locality is believed to be a characteristic of low-energy physical eigenstates, but is not itself sufficient to ensure that the resulting quantum chemistry problem is easy for quantum or classical simulation. Intuitively, this is due to the possibility of glassiness or frustration, where additional local interactions as L increases can drastically change the global ground-state.

Gaps. Another useful concept is the gap Δ between the ground-state and first excited state, and the related density of states near the ground-state. Many formal and empirical complexity results depend on the magnitude of this gap. For example, the area law in 1D can be proven for local Hamiltonians for constant Δ [22, 23], while the area law holds in all dimensions for constant Δ and a polynomial density of states near the ground-state [24].

Provably efficient algorithms in 1D. The power of area laws and bounded gaps is clearly seen in 1D, where there is a provably $\mathrm{poly}(L)$-time classical tensor network algorithm for the ground state energy problem, assuming the Hamiltonian has a constant gap and all interactions are nearest neighbor on a 1D spin chain [25, 26, 27, 28, 29] or on a tree with fractal dimension less than two [30]. This algorithm builds from techniques [23] used to prove the area law in 1D ground states in these cases to produce an estimate for the ground state energy (and an MPS description of the ground state) to precision ϵ with probability at least $1 - \delta$ in time $\mathrm{poly}(L)\,\mathrm{poly}(1/\epsilon)\,\mathrm{poly}(\log(1/\delta))$; the algorithm has been called a "rigorous" version of DMRG (although it is quite different from DMRG in certain respects). (Unfortunately, optimizing the ϵ-dependence of the algorithm has not been a focus of prior work).

Heuristic quantum states for gapless systems. As discussed above, the conjectured area law for critical quantum systems (e.g. ones with Fermi surfaces) contains logarithmic corrections. There are families of states that can be constructed to explicitly satisfy area laws with logarithmic corrections appropriate to critical states, e.g. the multiscale entanglement renormalization

ansatz (MERA) in 1D [31], and the branching MERA in higher dimensions [32]. Because of the mildness of the logarithmic correction, it is also common to use explicit area law states, but with polynomially growing overhead, to model critical states (e.g. DMRG with $\mathrm{poly}(L)$ bond dimension in 1D [33], or PEPS with $\mathrm{poly}(L)$ bond dimension in 2D [34]).

2.5 Sufficient conditions for the validity of the adiabatic approximation

Let $s(t)$ be a "schedule" function such that $s(0) = 0$ and $s(T_{\mathrm{ASP}}) = 1$, with T_{ASP} the total time of the evolution. Let $\Upsilon(s(t))$ denote the state of the quantum computer at time t. The adiabatic-state-preparation (ASP) protocol calls for the initial state $\Upsilon(0)$ to be the ground state Υ_0 of an initial Hamiltonian H_{init}, and for the state $\Upsilon(s)$ to subsequently evolve by the time-dependent Hamiltonian $H(s(t)) = (1 - s(t))H_{\mathrm{init}} + s(t)H_{\mathrm{final}}$ that interpolates from the initial Hamiltonian to the final Hamiltonian H_{final}. As $T_{\mathrm{ASP}} \to \infty$, it is guaranteed that $|\langle\Phi|\Psi_0\rangle| \to 1$, where $\Phi \equiv \Upsilon(1)$ is the end state of the ASP protocol and $\Psi_0 \equiv \Upsilon_0(1)$ is the ground state of H_{final}. For a desired success probability p_0 in QPE, one would like to choose the minimum value of T_{ASP} such that $|\langle\Phi|\Psi_0\rangle|^2 \geq p_0$; as discussed in Supplementary Note 3.5, the cost of QPE depends on the value of p_0 and taking $p_0 > 1/2$ is sufficient in our application.

Deducing rigorous analytical bounds on the total evolution time T_{ASP} involves two main challenges. First, one must gain a sufficiently precise understanding of the eigenstructure of the interpolating Hamiltonian $H(s)$; of particular importance is knowledge of a lower bound Δ on the ground-state gap that holds everywhere along the path. Finding such a bound is generally difficult, especially since situations where a numerical estimate of the ground state energy is most valuable are likely to be among the least amenable to analytical analysis. The numerics in the main text aim to compute such a bound, but this kind of numerical analysis will quickly become intractable as the system size increases. Second, even with complete knowledge of the spectral gap and other eigenstructure information, rigorous error bounds on $|\langle\Phi|\Psi_0\rangle|$ in the

literature are quite complex. An often-quoted heuristic condition for ASP success is $T_{\text{ASP}} \gg \max_{s,i} |\langle \Upsilon_i(s)|\, dH/ds\, |\Upsilon_0(s)\rangle| / (E_i(s) - E_0(s))^2$, where $\Upsilon_i(s)$ and $E_i(s)$ are the instantaneous eigenstates and eigenvalues of $H(s)$ [35]. Usually, the maximum is achieved at $i = 1$, in which case the above reads $T_{\text{ASP}} \gg \max_s \tau(s)$, motivating the "adiabatic time estimate" discussed in the main text. A weaker condition that can be rigorously proven for a large class of Hamiltonians is $T_{\text{ASP}} > K(\max_s \|dH/ds\|)\Delta^{-2} \log(\Delta^{-1})^{12}$ for some constant K, where $\|\cdot\|$ denotes the operator norm [36]; however, this bound is likely far from tight [37, 38].

These issues emphasize how, in most circumstances, it is expected that ASP will be applied heuristically, perhaps by trying increasingly larger values of T_{ASP}, as well as different choices of H_{init} and schedule function $s(t)$, until success is observed. For a particular choice of H_{init} and schedule, if the minimum eigenvalue gap Δ is exponentially small in L, ASP will fail for any choice of $T_{\text{ASP}} = \text{poly}(L)$.

On the other hand, the theoretical guarantees are strong enough to imply that $\Delta \geq 1/\operatorname{poly}(L)$ is sufficient to guarantee that ASP succeeds in time $\text{poly}(L)$. Thus, as EQA is an asymptotic proposition, whether EQA is typically achievable via ASP + QPE boils down simply to whether $\Delta \geq 1/\operatorname{poly}(L)$ and no efficient classical algorithm exists.

Supplementary Note 3 Supporting discussion and data for numerical experiments

3.1 Additional ansatz state preparation data

As a rough model for what happens when we try to improve the local fidelity for a large cluster such as FeMo-co, we performed DMRG calculations for the [2Fe-2S] cluster with two different oxidation states. We gradually increased the bond dimensions D from 1 to 2000 and we computed the infidelity and the energy error with respect to an almost exact DMRG state with a large bond dimension of 8000 as $I = 1 - |\langle\Psi_0(D)|\Psi_0(8000)\rangle|^2$ and $\epsilon = E(D) - E(8000)$.

Supplementary Figure 1 shows the computational effort (T defined in Supplementary Note 4.5) versus the inverse infidelity ($1/I$). The sublinear growth in the log-log scale means that the cost is poly($1/I$).

Similarly, Supplementary Figure 2 shows that the energy error is poly(I). Thus, improving the local fidelity efficiently improves the local energy. Assuming some product like structure in the global state, this form of the local improvement leads to a global overlap consistent with a good energy.

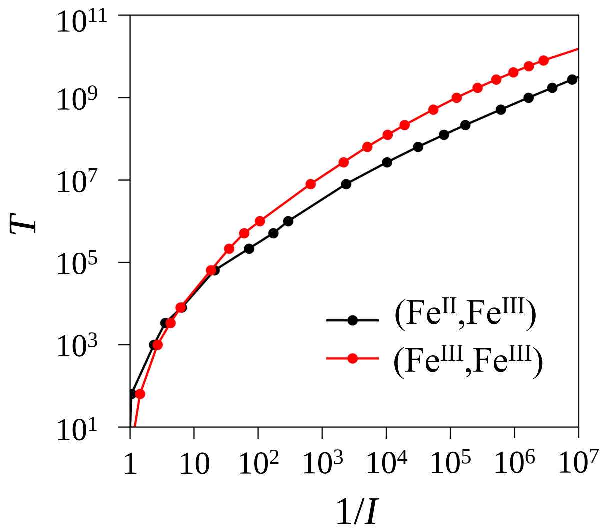

Supplementary Figure 1: Computational effort (T defined in Supplementary Note 4.5) with respect to the inverse infidelity ($1/I$).

3.2 Scaling of DMRG bond dimension in the Fe-S clusters

In the numerical study of FeS clusters, we performed DMRG calculations across a range of clusters (from [2Fe-2S] to the FeMo-co and P-clusters). Here we report on the DMRG bond dimension (in some cases, an estimate based on extrapolation) to reach constant $\bar{\epsilon}$ (here chosen as $\bar{\epsilon} = 10^{-3}$ Hartree per metal center). Supplementary Figure 3 shows the estimated DMRG bond dimension needed to reach this constant $\bar{\epsilon}$ as a function of the number of metal centers

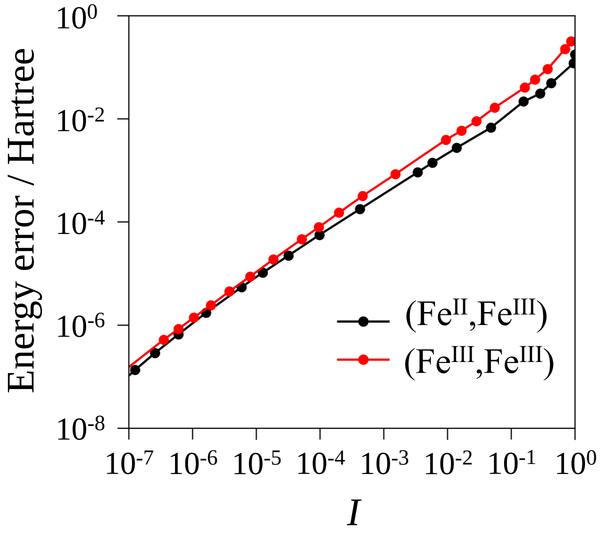

Supplementary Figure 2: Energy error versus the infidelity (I).

(2-8); the number of metals may be taken to be roughly proportional to L. Although there is some variation in the required bond dimension across clusters of the same nominal size (due to substantive differences in chemical composition, orbitals, and chemical structure amongst clusters with the same number of metals) we nonetheless can see that the bond dimension required for the 8Fe P^N and P^{ox} clusters is similar to that required for the smaller 4Fe clusters, and overall, there is a drop in slope between 2-4 and 4-8 metal ions. This reflects the relative similarity in cross-section when moving from 4 to 8 metal ions (and dissimilarity in cross-section when moving from 2 to 4 metal centers) and hints at a corresponding sub-volume (or area-law) behaviour of the entanglement, as might be expected in a chemical ground-state problem.

3.3 Additional discussion of adiabatic state preparation data

In the main text we showed estimated adiabatic state preparation times for a variety of different mean-field Hamiltonians and interacting Hamiltonians. Here we provide some additional interpretation of the associated figures. The technical definition of the mean-field Hamiltonians and interacting Hamiltonians is provided in Supplementary Note 5.

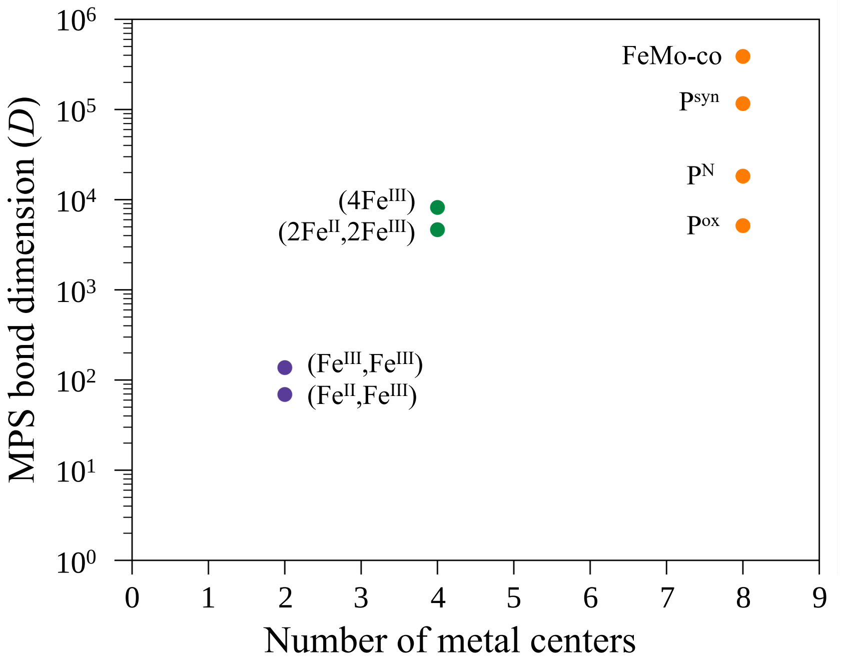

Supplementary Figure 3: Required bond dimension (D) of the spin-adapted MPS for an estimated energy error of 10^{-3} Hartree per metal center, with respect to the number of metal centers.

As discussed in the main text, the lowest energy mean-field (Slater determinant) state - the lowest eigenstate of a Kohn-Sham mean-field Hamiltonian - does not have good overlap with the desired final state, and this is correlated with a large $T^{\text{est}}_{\text{ASP}}$. The set of mean-field data corresponds to considering initial $H(0)$ whose ground-state is an excited Slater determinant (relative to the lowest energy Slater determinant). In practice, this can be done by adding a shift to the orbital energies to turn some excited-state orbitals into ground-state orbitals (see Supplementary Note 5). We consider over 100 such excited Slater determinants as initial states for the ASP procedure. Across these initial states, $T^{\text{est}}_{\text{ASP}}$ has a good correlation with the inverse weight $|\langle\Upsilon_0(0)|\Psi_0\rangle|^2$.

In addition, an entirely different set of initial states were obtained by choosing initial interacting Hamiltonians defined in active spaces $n_{\text{act}} < 12$ (definition in Supplementary Note 5). (For $n_{\text{act}} = 12$, determining the initial state would correspond to finding the ground-state of the full problem). However, the associated $T^{\text{est}}_{\text{ASP}}$ show the same inverse weight behaviour as those from the mean-field initial Hamiltonians, and the data from the two show a good correlation on the same plot. This suggests that in this problem the adiabatic state preparation time correlates well with the inverse initial weight, at least for this choice of adiabatic schedule.

We also see a linear trend when plotting $1/(\min_s \Delta(s))$ against $|\langle\Upsilon_0|\Psi_0\rangle|^2$ in Supplementary Figure 4. This shows that the minimum ground-state gap ($\min_s \Delta(s)$) is the main factor governing the behaviour of the adiabatic estimates.

3.4 Time-dependent simulations of adiabatic state preparation

When discussing the relationship between T_{ASP} (for 75% final weight) and its adiabatic estimate $T^{\text{est}}_{\text{ASP}}$ in the main text, we concluded that ratio was $O(1)$, based on 17 time-dependent simulations of ASP shown in Figure 2B of the main text. For these ASP simulations, we used 17 mean-field initial Hamiltonians and the linear-interpolated adiabatic path (definition in Supple-

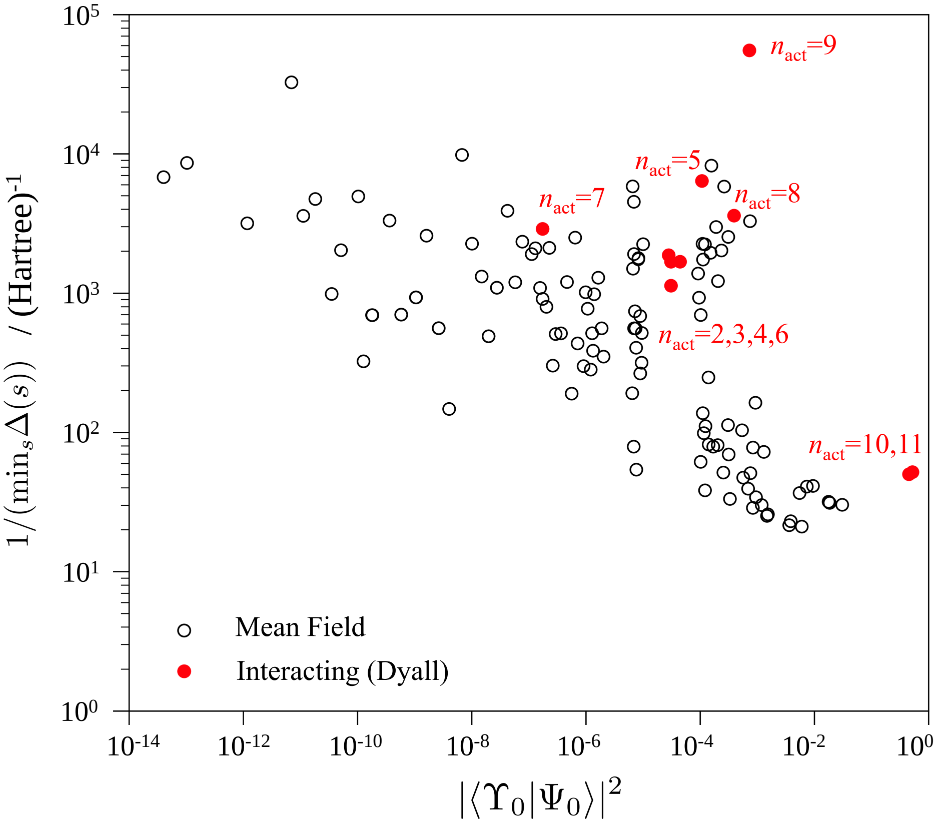

Supplementary Figure 4: Inverse minimum ground-state gap ($1/(\min_s\Delta(s))$) along the adiabatic preparation path versus the weight of the initial ground state (Υ_0) in the final ground state. (Ψ_0)

mentary Note 5). The weights of the 17 initial states ($\Upsilon_0(0)$) in the desired final ground state (Ψ_0) ranged from 4×10^{-9} to 3×10^{-2}.

Here we show one example of these time-dependent ASP simulations, corresponding to a mean-field initial ground state (Υ_0) with weight $|\langle\Upsilon_0(0)|\Psi_0\rangle|^2 = 2 \times 10^{-4}$. The adiabatic time estimate ($T^{\text{est}}_{\text{ASP}}$) for this initial state is $660\ E_h^{-1}$. We numerically propagated the wavefunction with a time-step $\Delta t = 0.01 E_h^{-1}$, via

$$|\Upsilon(s(t+\Delta t))\rangle = e^{-iH(s(t+\Delta t/2))\Delta t}|\Upsilon(s(t))\rangle. \tag{1}$$

The action of the exponential was approximated by the Runge-Kutta 4th order method. Sup-

plementary Figure 5 shows the weight of the adiabatically prepared state ($\Upsilon(s)$) as a function of the "schedule" function $s(t)$ (where $s(0) = 0$ and $s(T_{\mathrm{ASP}}) = 1$). In this example, 75% final weight is achieved with $T_{\mathrm{ASP}}/T_{\mathrm{ASP}}^{\mathrm{est}} \approx 2$.

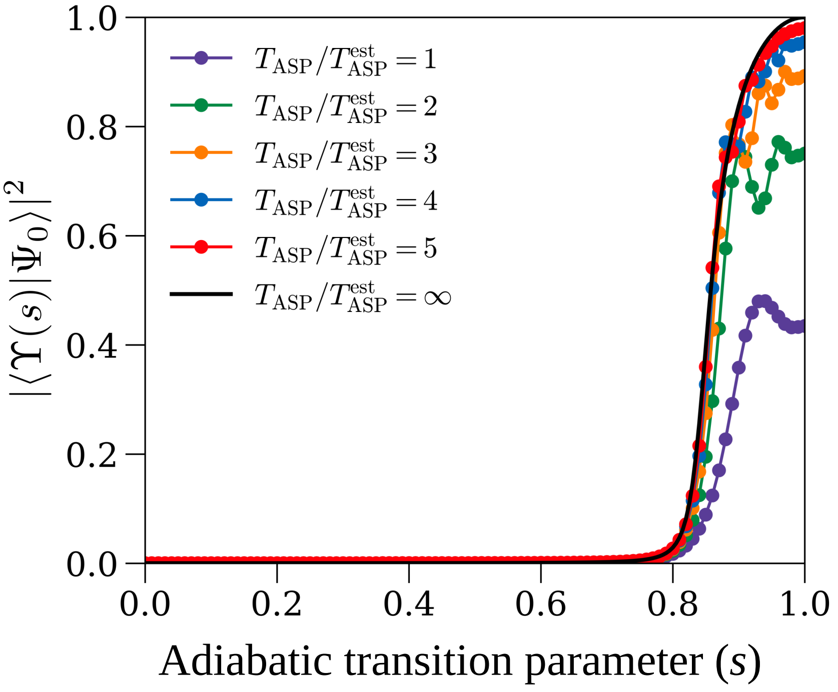

Supplementary Figure 5: Weight $|\langle \Upsilon(s)|\Psi_0\rangle|^2$ of the adiabatically prepared state as a function of s for different total ASP time T_{ASP} in time-dependent ASP simulations of a [2Fe-2S] model.

3.5 Evolution time for quantum phase estimation

We now describe the method we use for quantifying the evolution time required in performing QPE. By evolution time we mean the sum of all time t's in the unitary operators of the form e^{-iHt} that we need to implement. We assume we have an initial state $|\Phi\rangle$ satisfying $|\langle\Phi|\Psi_0\rangle|^2 = p_0 > 1/2$. We want to estimate the ground state energy to within error ϵ with probability at least 0.90. In a single run of QPE, if we want the success probability to be at least p_s ($p_s < p_0$ because we are only considering the success probability of a single run), then according to the

analysis in Section 5.2.1 in Ref. [39], the required evolution time is

$$T = \frac{\pi}{\epsilon(1 - p_s/p_0)}. \tag{2}$$

Here by success we mean that the estimate is ϵ-close to the ground state energy. Now we run QPE multiple times and take the median to boost the success probability. If more than half of the trials are successful, then the median will be ϵ-close to the ground state energy. Therefore the boosted success probability is at least

$$p_{\text{boosted}} = \sum_{m=(n_{\text{trial}}+1)/2}^{n_{\text{trial}}} \binom{n_{\text{trial}}}{m} p_s^m (1 - p_s)^{n_{\text{trial}}-m}. \tag{3}$$

We then choose the minimal n_{trial} that ensures $p_{\text{boosted}} \geq 0.90$. The evolution time required for QPE is then

$$n_{\text{trial}} T = \frac{\pi n_{\text{trial}}}{\epsilon(1 - p_s/p_0)}. \tag{4}$$

In the above method, p_s can be optimized to minimize the required evolution time. Also here we have only considered the simplest version of QPE, where the energy register is initialized to be the equal superposition of all bit strings. A better initialization using a resource state that minimizes the variance [40, 41], or the Kaiser window [42, 43], should help reduce the required evolution time. In the main text, a contrast is drawn between the QPE evolution time and the time for ASP; thus any further improvement in the QPE evolution time only serves to more strongly highlight the time cost of ASP.

3.6 Overview of systems chosen for scaling of classical heuristics

In the discussion of scaling of classical heuristics in the main text, we carried out illustrative calculations on large molecular problems (alkane chains and a protein fragment) as well as lattice problems relevant to correlated materials. Here we supply further background discussion regarding the relevance of these problems.

The molecular systems are representative of molecules encountered in organic and biological chemistry. The defining characteristic is the "single-reference" nature of the ground-state, where there is assumed to be good local overlap with a mean-field Slater determinant (although the global overlap still decays like $e^{-\alpha L}$). In this setting, classical heuristics such as the coupled cluster method (see Supplementary Note 6) are often successful. The single-reference character is usually lost at points in a reaction where bonds are being broken or being formed. However, the non-trivial behaviour in the ground-state is then expected to be confined to a small number of atoms in an "active" region where the reaction is occurring. This is the basis of active space models, as for example, used to study the Fe-S metalloclusters embedded in the larger nitrogenase enzyme.

The second set of model problems is representative of models of correlated materials. The Heisenberg model is a model of a quantum magnet. On unfrustrated lattices, such as the cubic lattice, one can obtain exact results (up to sampling error) using quantum Monte Carlo due to the special sign-free structure of the Hamiltonian. This was used to generate exact data for this problem (see Supplementary Note 7.3). However, the tensor network calculations we presented on the same model do not take advantage of the sign-free structure; and further, the Heisenberg model is gapless, while the particular PEPS tensor network is designed to capture area law states. From this perspective, the 3D Heisenberg model might be expected to be hard for tensor network heuristics. However, as seen from the data, calculations on up to 1000 sites can be done with close to $O(L)$ cost for $\bar{\epsilon} \sim O(10^{-3})$. More interesting physics can be seen in quantum magnets on frustrated lattices or with frustrating interactions. Although larger bond dimensions are typically required to reach the same accuracy as in unfrustrated models, tensor network calculations on frustrated models can also reach sufficient accuracy to resolve the interesting physics, with poly(L) cost, see e.g. [44, 45].

The 2D Hubbard model is a paradigmatic model of correlated materials. Similarly to as

in the 2D Heisenberg model, the half-filled point in the phase diagram can be simulated by sign-free quantum Monte Carlo methods. However, away from half-filling the phase diagram is challenging, with the 1/8 doping point considered one of the most challenging points. For narrow lattices, numerically exact results can be generated by DMRG, and we used this to generate exact benchmark data for the 4×8 and 4×16 lattices. However, the PEPS calculations reported in the main text do not in practice encounter the same exponential scaling with width as DMRG and can thus be used on wider lattices. Note that the specific 2D Hubbard model calculations do not represent the largest or most accurate tensor network calculations performed in this model; instead they were performed in such a way that the energies could be directly compared to DMRG calculations so that the error scaling could be assessed. For a more detailed study of the 2D Hubbard model using tensor networks, including the physics of competing low energy states, see e.g. Ref. [46].

We also note that tensor networks are by no means the only technique that can successfully treat strongly correlated models. For some alternatives in frustrated spin models, see e.g. Ref. [47]; for some other techniques used in challenging parts of the 2D Hubbard model phase diagram, see e.g. Ref. [46, 48].

Some of the techniques used in strongly correlated lattice models have now been generalized to ab initio Hamiltonians. For some examples, see Ref. [16]. With respect to tensor networks as used in the main text, the density matrix renormalization group and tree tensor networks have a long history of use in ab initio quantum chemistry [9, 49, 50, 51, 52]. PEPS have not yet been used in ab initio quantum chemistry, but generalizations towards ab initio models (e.g. calculations for fermionic continuum problems [53] and using long-range interactions [54]) have started to appear. Note that the physical principle of locality exploited by tensor networks is central to other heuristics for strongly correlated problems, most notably quantum embedding methods, such as dynamical mean-field theory (DMFT) [55] and density matrix embedding

theory (DMET) [56]. Rather than target an ansatz for the global quantum state, these methods seek to assemble the relevant physical observables (e.g. Green's functions and density matrices) from calculations on local subsystems. They can be routinely applied to ab initio quantum chemical Hamiltonians [57, 58], and we use LIBDMET [59] to perform the DMET calculations in this work. In Supplementary Figure 6**A**, we show the timings for ab initio quantum chemistry DMET calculations on hydrogen cubes of size up to $10 \times 10 \times 10$ (minimal basis). This may be considered an ab initio analog of the Hubbard model. We see that the time per hydrogen atom increases only polynomially with system size. (Note that the time has two components: the time to solve the correlated DMET impurity problems, which is approximately constant per atom, and the time to set up the Hamiltonian for the DMET impurity problem for each atom, which grows polynomially with system size). In Supplementary Figure 6**B**, we show DMET calculations with similar settings for a series of 2D hydrogen lattices of size 4×4 to 4×16, where we see that the absolute error achieved is close to 2 milliHartrees per atom. The precision scaling of embedding methods is currently not well characterized, but for some data and discussion of scaling of accuracy of local expectation values with embedding cluster size, see e.g. Refs. [60, 61]. These works provide numerical evidence of the convergence of local expectation values with cluster size (when appropriately evaluated) for a limited number of systems.

3.7 Error scaling and cost of local coupled cluster theory

In the main text, we showed that the computational effort to reach a given precision (error) is polynomial in the inverse precision. The computational time metric used was L^{2m+2} (here L is the number of orbitals), where m is the excitation level ($= 2$ for CCSD, 3 for CCSDT etc.). This is the cost scaling of the most expensive tensor contraction that dominates the computation in these methods.

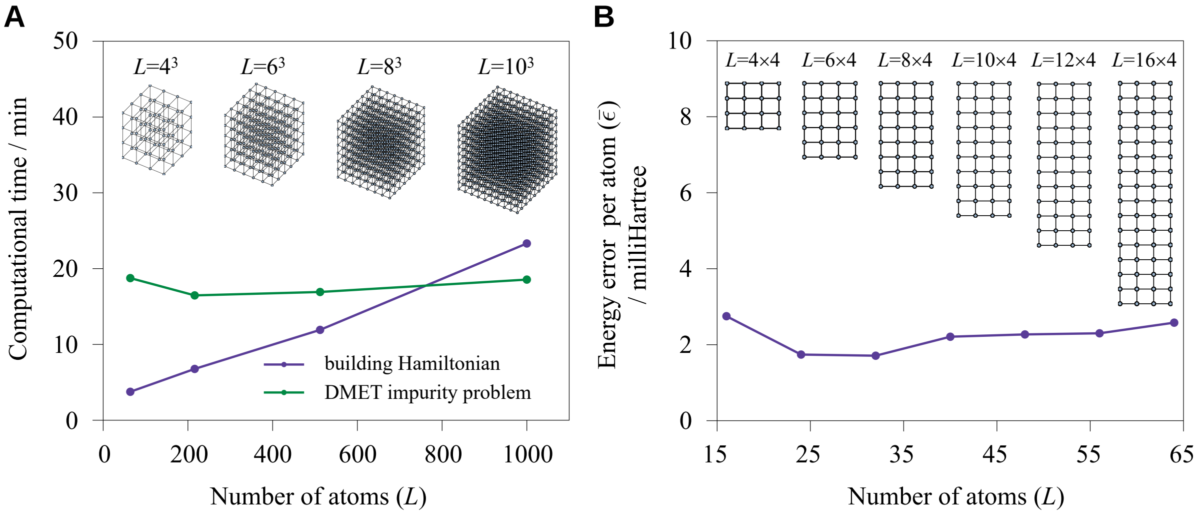

Supplementary Figure 6: Ab initio calculations on hydrogen cubes and 2D lattices. The calculations are performed at a H-H separation of 1 Å. **A,** timing per atom for hydrogen cubes, **B,** error per atom for 2D lattices, compared against near-exact DMRG. **A,** using $2 \times 2 \times 2$ fragments, **B,** using 2×2 fragments.

In a local coupled cluster theory as shown in the main text, the time is reduced to $O(L)f(m)$, where $f(m)$ depends on the number of local orbitals L_{loc} involved in the excitation, and is $\sim L_{\text{loc}}^{2m+2}$. Local versions of CC beyond CCSD(T) have not yet been implemented, however, assuming the same conjectured error scaling as without the local heuristic, the anticipated total cost (for problems where CC is a good heuristic) for local CC methods is $O(L)\text{poly}(1/\epsilon)$.

Supplementary Note 4 Technical details: electronic ground-state calculations for the Fe-S clusters

4.1 Active space models

In our Fe-S simulations, we used active space models which capture the important aspects of the electronic structure of these systems. In particular, we used active space models from previous studies for the [2Fe-2S] and [4Fe-4S] [62], P-cluster [63], and FeMo-cofactor [64] systems. Detailed information about these models can be found in Refs. [62, 63, 64]. In this subsection,

we briefly summarize the approximations used to construct the electronic models.

Optimized BS-DFT geometries were used for the synthetic [2Fe-2S] and [4Fe-4S] clusters [65] while X-ray crystal structures were used for the P-clusters [66, 67] and the FeMo-cofactor [68]. All the active space models were constructed from split-localized [69, 70] natural orbitals from high-spin unrestricted DFT wavefunctions incorporating scalar relativistic effects. Calculations on the [2Fe-2S] and [4Fe-4S] clusters used the BP86 functional [71, 72], TZP-DKH basis [73], and the spin-free exact two-component (sf-X2C) Hamiltonian [74, 75, 76]. Calculations on the P-clusters used the BP86 functional [71, 72], def2-SVP basis [73], and the sf-X2C Hamiltonian [74, 75, 76]. Calculations on the FeMo-co clusters used the B3LYP functional, [77, 78, 79] TZP-DKH basis [73] for Fe, S, and Mo, def2-SVP basis [73] for the other atoms (C, H, O, and N), and the sf-X2C Hamiltonian [74, 75, 76]. In addition, the calculations on the P-clusters and the FeMo-cofactor used the conductor like screening model (COSMO) [80] with a dielectric constant $\epsilon = 4.0$ to mimic the protein environment.

Finally, all the active space models were constructed from the full valence space of the Fe $3d$, S $3p$, and bonding ligand orbitals around each metal atom. The active space model for the FeMo-cofactor also contained the additional full valence space of the Mo $4d$ and central C $2s$ and $2p$. The resulting active space models consisted of CAS(30e,20o) for (Fe^{III}, Fe^{III}), CAS(31e,20o) for (Fe^{II}, Fe^{III}), CAS(54e,36o) for ($2\,Fe^{II}, 2\,Fe^{III}$), CAS(52e,36o) for ($4\,Fe^{III}$), CAS(108e,71o) for P^{syn}, CAS(114e,73o) for P^{N}, CAS(120e,77o) for P^{ox}, and CAS(113e,76o) for the FeMo-cofactor.

4.2 Initial guesses for DMRG calculations

There are many local electronic minima in the energy landscape of ansatz approximations to the P-cluster and FeMo-cofactor ground-state wavefunctions. To reach the correct ground-state, it is important to start from a good initial guess. We used a special DMRG initialization procedure

presented in earlier studies [62, 63]. In this subsection, we briefly summarize the procedure.

As an initial guess to the spin-adapted DMRG calculation, we first performed a spin-projected (SP)-MPS calculation [62]. This was initialized by spin-projecting a broken-symmetry determinant chosen to represent the spin-coupling pattern shown in Supplementary Figure 7 with the total spin, total charge, and the oxidation number states of the metal cluster as summarized in Supplementary Table 1. The resulting spin-projected MPS was further optimized up to a bond dimension of 50, and then fed to the spin adapted DMRG calculation.

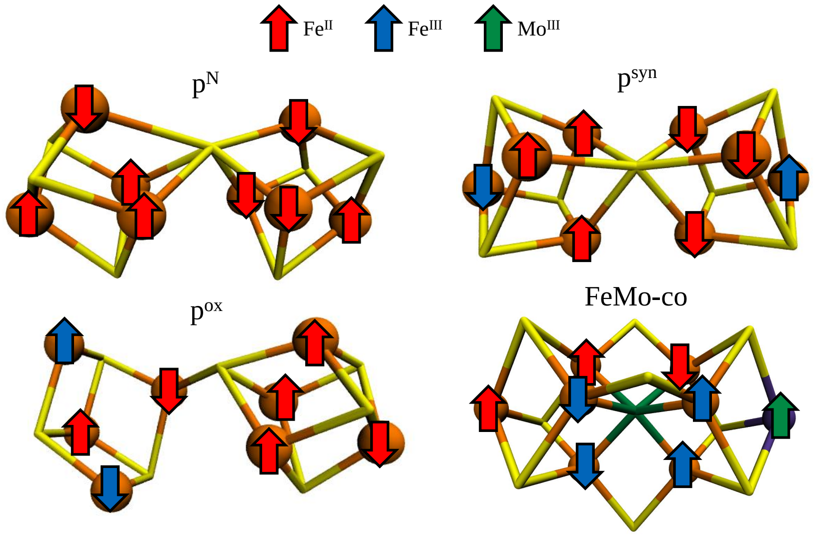

Supplementary Figure 7: Spin-coupling pattern of the broken symmetry guess for the electronic ground states reported in previous theoretical studies [63, 81].

Supplementary Table 1: Total spin and total charge of the metal clusters and the oxidation number of each metal atom for the broken symmetry guess of the electronic ground state.

	Total spin	Total charge	Oxidation number
p^{N}	$S = 0$	-4	$4Fe^{II}\uparrow\ 4Fe^{II}\downarrow$
p^{syn}	$S = 0$	-2	$Fe^{III}\uparrow\ Fe^{III}\downarrow\ 3Fe^{II}\uparrow\ 3Fe^{II}\downarrow$
p^{ox}	$S = 4$	-4	$Fe^{III}\uparrow\ Fe^{III}\downarrow\ 4Fe^{II}\uparrow\ 2Fe^{II}\downarrow$
FeMo-co	$S = 1.5$	-3	$2Fe^{III}\uparrow\ 2Fe^{III}\downarrow\ 2Fe^{II}\uparrow\ Fe^{II}\downarrow\ Mo^{III}\uparrow$

4.3 Extrapolations for the total DMRG energies

We extrapolated the ground-state DMRG energy as a function of discarded weight, see e.g. Ref. [82]. Except for FeMo-co, the MPS energies at smaller bond dimension were obtained from "reverse sweep schedule" DMRG calculations, starting from the converged MPS with the largest bond dimension. We excluded the energy with the largest bond dimension, which was not fully converged, from the energy extrapolations.

The above extrapolations indicate that for the [4Fe-4S] clusters, the extrapolated exact energy is between 3-5 mE_h (extrapolation distance) below the lowest DMRG variational energy, approximately 1 mE_h per Fe. It is difficult to estimate the error of the extrapolated energy itself, but it can conservatively be taken to be some fraction of the extrapolation distance. (Note that the designation of chemical accuracy ($\sim$ 1kcal/mol) was originally intended to refer to thermochemical energy *differences*, e.g. heats of formation and ionization potentials, mainly in small molecules [83]). In the absence of a specific chemical transformation to discuss (which would involve changing the electronic structure of only a part of the system) the more relevant metric on the total energy is often $\bar{\epsilon}$ rather than ϵ, because chemical reactions usually involve changes in a small part of the total system.

In the P- and FeMo-co clusters, the extrapolation distance is somewhat larger, ranging from about 10 mE_h (P^{ox}) to more than 80 mE_h in FeMo-co (approximately 1-10 mE_h per metal atom), reflecting the greater complexity. Although the extrapolation distance in the FeMo-cofactor is larger than desired, the convergence of the energy remains superpolynomial in $1/\epsilon$ (see Supplementary Figure 9) as for the other clusters, and the extrapolation is sufficiently systematic to provide some confidence in the extrapolated energy.

Supplementary Table 2: Total DMRG energies in E_h for the electronic ground states of the Fe-S clusters. D denotes the bond dimension in the spin-adapted MPS ansatz.

D	Discarded weight	Energy / E_h	D	Discarded weight	Energy / E_h
	2Fe(II)-2Fe(III)			4Fe(III)	
1000	3.73×10^{-4}	-8432.789966	1000	3.03×10^{-4}	-8432.606085
2000	2.01×10^{-4}	-8432.798444	2000	1.78×10^{-4}	-8432.615484
3000	1.34×10^{-4}	-8432.801780	3000	1.29×10^{-4}	-8432.619229
4000	9.76×10^{-5}	-8432.803590	4000	1.01×10^{-4}	-8432.621279
5000	7.43×10^{-5}	-8432.804722	5000	8.18×10^{-5}	-8432.622578
6000	5.84×10^{-5}	-8432.805500	6000	6.70×10^{-5}	-8432.623476
7000	4.65×10^{-5}	-8432.806069	7000	5.44×10^{-5}	-8432.624094
8000	3.50×10^{-5}	-8432.806502	∞		-8432.6287(9)
∞		-8432.8084(4)			
	P^{syn}			P^{N}	
1000	5.08×10^{-4}	-17833.977588	1000	4.17×10^{-4}	-17492.190311
1500	4.41×10^{-4}	-17833.989684	1500	3.40×10^{-4}	-17492.200594
2000	3.88×10^{-4}	-17833.996979	2000	2.89×10^{-4}	-17492.206725
2500	3.50×10^{-4}	-17834.001975	2500	2.51×10^{-4}	-17492.210894
3000	3.21×10^{-4}	-17834.005658	3000	2.23×10^{-4}	-17492.213953
3500	2.95×10^{-4}	-17834.008503	3500	2.00×10^{-4}	-17492.216294
4000	2.74×10^{-4}	-17834.010764	4000	1.82×10^{-4}	-17492.218146
4500	2.53×10^{-4}	-17834.012610	4500	1.63×10^{-4}	-17492.219644
5000	2.27×10^{-4}	-17834.014051	5000	1.36×10^{-4}	-17492.220847
∞		-17834.049(7)	∞		-17492.239(4)
	P^{ox}			FeMo-co	
500	4.06×10^{-4}	-17489.880043	2000	4.53×10^{-4}	-22140.21120
1000	2.60×10^{-4}	-17489.895112	3000	3.77×10^{-4}	-22140.24039
1500	1.94×10^{-4}	-17489.901003	4000	3.19×10^{-4}	-22140.25652
2000	1.57×10^{-4}	-17489.904229	4500	3.02×10^{-4}	-22140.26141
2500	1.34×10^{-4}	-17489.906285	5000	2.86×10^{-4}	-22140.26562
3000	1.17×10^{-4}	-17489.907715	5500	2.69×10^{-4}	-22140.26995
3500	1.03×10^{-4}	-17489.908781	6000	2.60×10^{-4}	-22140.27335
4000	8.70×10^{-5}	-17489.909590	6500	2.55×10^{-4}	-22140.27633
∞		-17489.919(2)	7000	2.47×10^{-4}	-22140.27881
			∞		-22140.36(2)

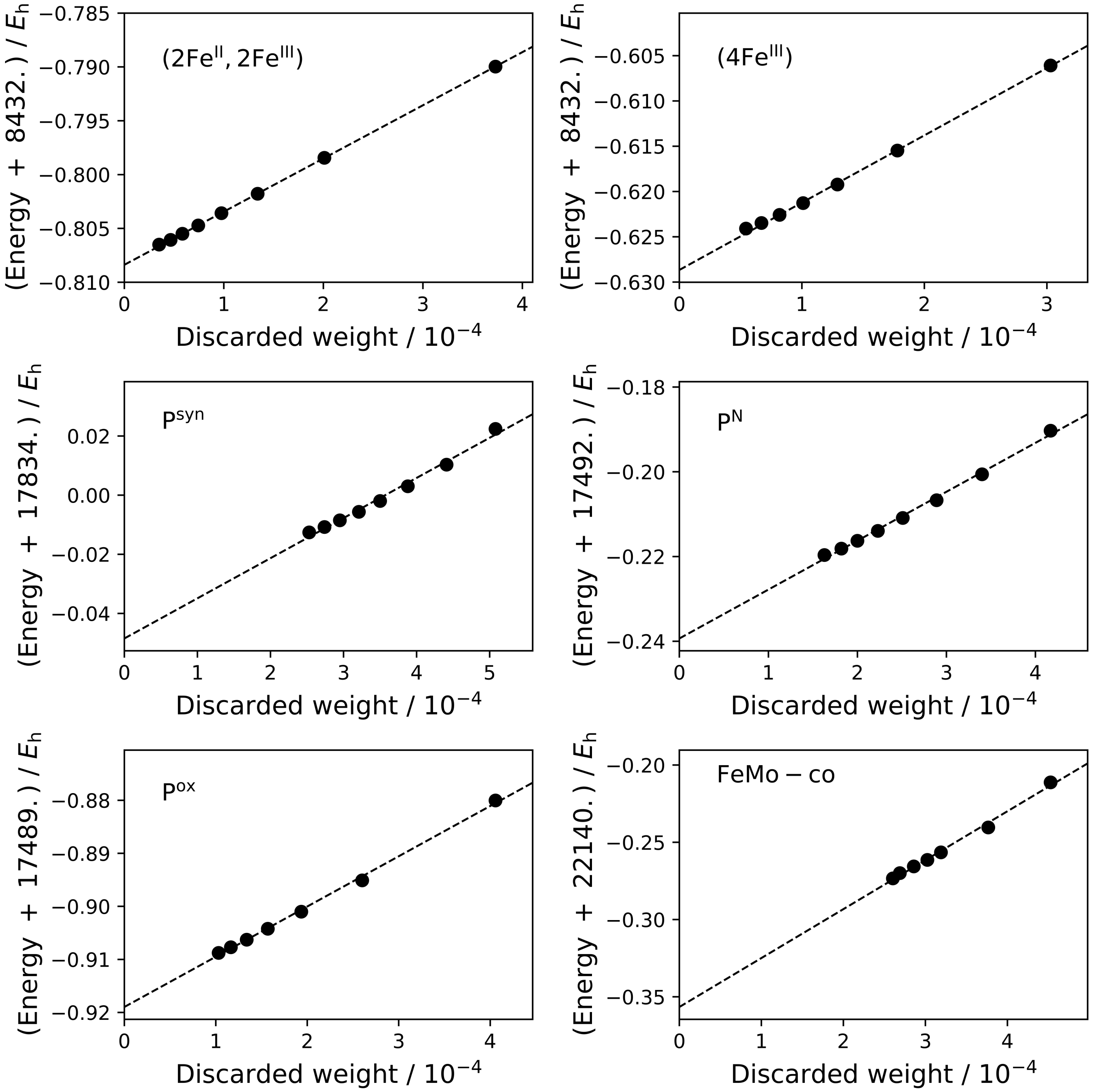

Supplementary Figure 8: DMRG energy in E_h of the Fe-S clusters versus the discarded weight. The dashed lines represent the best fit straight lines which are extrapolated to zero discarded weight to obtain an estimate of the exact energy.

4.4 Extracting the largest weight determinant and configuration state function

We extracted the (approximately) largest weight Slater determinant (Φ_D) and configuration state function (Φ_{CSF}) from the optimized MPS ($\Psi_0(D)$) with bond dimension D, by gradually com-

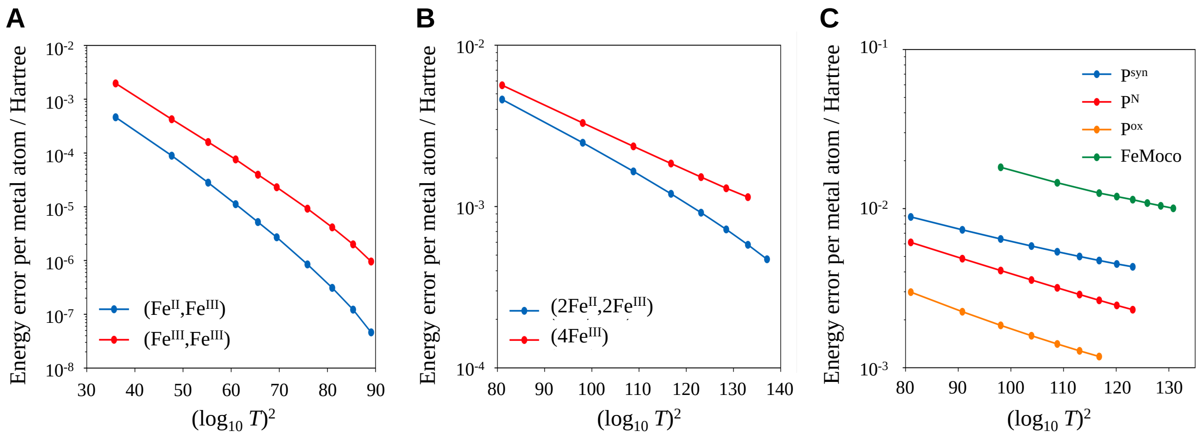

Supplementary Figure 9: Convergence of energy error per metal atom of the Fe-S clusters with respect to computational effort (T defined in Supplementary Note 4.5). The observed empirical $\log(1/\bar{\epsilon}) \sim (\log T)^2$ relation corresponds to superpolynomial $1/T$ error convergence. The errors are obtained with respect to the exact FCI energies for the [2Fe-2S] systems (**A**), and extrapolated DMRG energies in the other clusters. (**B** and **C**)

pressing the MPS down to a bond dimension of one. This provides an approximate way to obtain a Slater determinant (from compressing a non-spin-adapted MPS) or configuration state function (CSF) (spin-adapted MPS) that has the largest weight in the MPS. (It should be noted that this procedure is not guaranteed to find the absolutely largest weight determinant or CSF, since the compression is done as a sweep rather in a globally optimal way.) The results are summarized in Supplementary Table 3.

4.5 Computational time metric

The computational cost of the DMRG quantum chemistry algorithm is $O(D^3L^3) + O(D^2L^4)$. For the DMRG calculations of the Fe-S clusters, the bond dimension (c.a. > 1000) is much larger than the number of active space orbitals (c.a. < 100), thus the cubic term dominates. The metric of DMRG computational time is $T = D^3$ in Fig. 2 of the main text; this factorizes out the system size dependence of the Hamiltonian, allowing for a simpler comparison between the different systems for quantities controlled by D (such as the error). Supplementary Figure 10

Supplementary Table 3: Overlap of the largest weight Slater determinant ($|\langle\Phi_D|\Psi_0\rangle|$) and overlap of the largest weight configuration state function ($|\langle\Phi_{\mathrm{CSF}}|\Psi_0\rangle|$) in the MPS wavefunction with bond dimension D; the weights plotted in the main text correspond to the squared overlap. The $D=\infty$ overlap uses an empirical extrapolation form $S(D) = c/D + S(\infty)$.

D	Overlap (S)		D	Overlap (S)	
	CSF	DET		CSF	DET
	2Fe(II)-2Fe(III)			4Fe(III)	
2000	1.66×10^{-2}	3.54×10^{-3}	2000	7.12×10^{-2}	2.15×10^{-2}
3000	1.61×10^{-2}	3.44×10^{-3}	3000	6.89×10^{-2}	2.08×10^{-2}
4000	1.58×10^{-2}	4.99×10^{-3}	4000	6.75×10^{-2}	2.03×10^{-2}
5000	1.56×10^{-2}	4.93×10^{-3}	5000	6.65×10^{-2}	2.01×10^{-2}
6000	1.54×10^{-2}	4.88×10^{-3}	6000	6.59×10^{-2}	1.99×10^{-2}
7000	1.53×10^{-2}	4.84×10^{-3}	7000	6.54×10^{-2}	1.97×10^{-2}
∞	1.47×10^{-2}	4.65×10^{-3}	∞	6.34×10^{-2}	1.90×10^{-2}
	$\mathrm{P^{syn}}$			$\mathrm{P^{N}}$	
1000	4.51×10^{-3}	1.97×10^{-3}	1000	2.44×10^{-2}	1.10×10^{-2}
1500	4.32×10^{-3}	2.05×10^{-3}	1500	2.05×10^{-2}	1.03×10^{-2}
2000	4.09×10^{-3}	2.04×10^{-3}	2000	1.80×10^{-2}	9.73×10^{-3}
2500	3.93×10^{-3}	2.02×10^{-3}	2500	1.62×10^{-2}	9.22×10^{-3}
3000	3.80×10^{-3}	2.00×10^{-3}	3000	1.49×10^{-2}	8.81×10^{-3}
3500	3.69×10^{-3}	1.99×10^{-3}	3500	1.39×10^{-2}	8.48×10^{-3}
4000	3.59×10^{-3}	1.98×10^{-3}	4000	1.32×10^{-2}	8.24×10^{-3}
4500	3.51×10^{-3}	1.97×10^{-3}	4500	1.27×10^{-2}	8.07×10^{-3}
5000	3.45×10^{-3}	1.96×10^{-3}	5000	1.23×10^{-2}	7.95×10^{-3}
∞	3.15×10^{-3}	1.92×10^{-3}	∞	8.63×10^{-3}	6.83×10^{-3}
	$\mathrm{P^{ox}}$			FeMo-co	
500	5.66×10^{-3}	1.94×10^{-3}	6000	3.68×10^{-4}	
1000	5.33×10^{-3}	1.78×10^{-3}	7000		9.88×10^{-5}
1500	5.33×10^{-3}	1.70×10^{-3}			
2000	5.08×10^{-3}	1.65×10^{-3}			
2500	5.00×10^{-3}	1.61×10^{-3}			
3000	4.94×10^{-3}	1.57×10^{-3}			
3500	4.90×10^{-3}	1.55×10^{-3}			
4000	4.86×10^{-3}	1.53×10^{-3}			
∞	4.72×10^{-3}	1.44×10^{-3}			

shows the strong linear relation between this time metric and the actual CPU computational time in seconds.

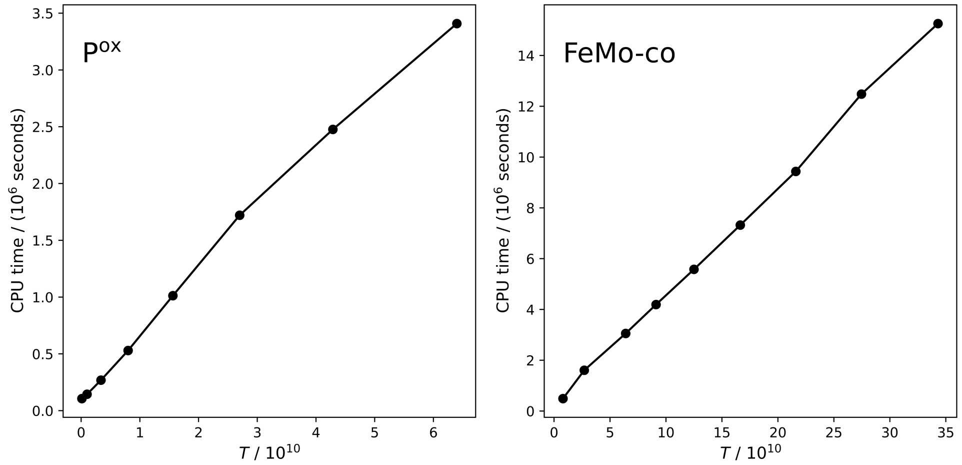

Supplementary Figure 10: Linear relation between the DMRG time metric used in the main text ($T = D^3$) and the actual computational time (seconds) for a DMRG sweep with the bond dimension D.

4.6 Additional convergence data for the [2Fe-2S] clusters

As the exact FCI data is available for the [2Fe-2S] clusters, in Supplementary Figure 11 we show some additional plots of DMRG convergence with bond dimension, for various quantities.

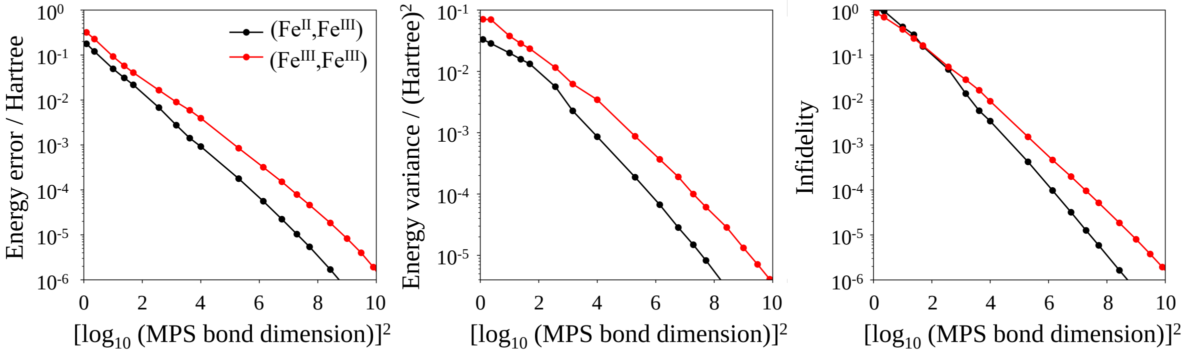

Supplementary Figure 11: Convergence of the energy error ($E_{\mathrm{DMRG}} - E_{\mathrm{FCI}}$), the energy variance ($\langle \Phi_{\mathrm{DMRG}} | (H - E_{\mathrm{DMRG}})^2 | \Phi_{\mathrm{DMRG}} \rangle$), and the infidelity ($1 - |\langle \Phi_{\mathrm{DMRG}} | \Psi_{\mathrm{FCI}} \rangle|^2$) with the MPS bond dimension (D) for the [2Fe-2S] models.

Supplementary Note 5 Technical details: Adiabatic state preparation

5.1 CAS model for adiabatic state preparation

We examined ASP for the singlet ground state of the [2Fe-2S] cluster, (Fe^{III}, Fe^{III}). Because the corresponding classical simulations are very expensive (due to the need to prepare states very slowly) we constructed smaller active space models. We used a Kohn-Sham orbital active space derived from DFT calculations using the BP86 functional and the scalar relativistic X2C Hamiltonian using the TZP-DKH basis. From these calculations, we obtained a CAS(14e,12o) model and verified that it produced similar chemistry to that of the CAS(30e,20o) model used in the FCI calculations. The 12 orbitals consist of the ten $3d$ orbitals of the irons and two out-of-plane $3p$ orbitals of the bridging sulfurs; "similar chemistry" here means that the Hamiltonian yields the same order of low-lying spin states, similar shapes of the natural orbitals and similar natural orbital occupations.

The Hamiltonian of this CAS(14e,12o) model can be written as

$$H = \sum_{pq} h^{\mathrm{C}}_{pq} \hat{E}_{pq} + \frac{1}{2} \sum_{pqrs} (pq|rs)(\hat{E}_{pq}\hat{E}_{rs} - \delta_{qr}\hat{E}_{ps}) + K_{\mathrm{C}}, \tag{5}$$

with

$$\hat{E}_{pq} = \hat{a}^{\dagger}_{p\alpha}\hat{a}_{q\alpha} + \hat{a}^{\dagger}_{p\beta}\hat{a}_{q\beta}, \tag{6}$$

$$h^{\mathrm{C}}_{pq} = h_{pq} + \sum_{I} (2(pq|II) - (pI|Iq)), \tag{7}$$

$$K_{\mathrm{C}} = 2\sum_{I} h_{II} + \sum_{IJ} (2(II|JJ) - (IJ|JI)), \tag{8}$$

where the orbital indices $\{p, q, r, s\}$ are used for the 12 orbitals in the active space, $\{I, J\}$ are used for orbitals in the core (doubly occupied) space, and α, β are the spin indices. h_{pq} and $(pq|rs)$ are the one-electron and two-electron integrals, respectively; h^{C}_{pq} is the effective one-electron integral (that includes the interactions with the core orbitals) and K_{C} is the core energy.

5.2 Initial Hamiltonian for the adiabatic state preparation

The ASP protocol prepares the state Φ_0 by starting in the ground state of an easy-to-prepare Hamiltonian. We tested two types of initial Hamiltonians.

Mean-field Hamiltonian: We prepared a mean-field initial Hamiltonian defined as the sum of the Kohn-Sham Fock operators. In addition, we added an energy shift (δ) to target a specific mean-field determinant as the ground state and to introduce a non-trivial energy gap between the ground and first excited state. Using the same notation for the orbital indices introduced in Supplementary Equation (5), the mean-field Hamiltonian is

$$H_{\text{Fock}} = \sum_p (\epsilon_p + \delta(n_p))\hat{E}_{pp} + K_{\text{C,Fock}}, \tag{9}$$

where ϵ_p is the Kohn-Sham orbital energy of the pth orbital and $K_{\text{C,Fock}} = 2\sum_I \epsilon_I$ is the energy from the core orbitals. The shift δ is a function of the occupation number (n_p) of the pth orbital (n_p corresponds to the occupations in the targeted mean-field determinant). We used $\delta(n_p = 2) = 0$ and $\delta(n_p = 0) = 0.5$ (Hartree). We find that the energy shift ensures that the targeted mean-field state is the ground state and the initial energy gap is larger than 0.35 Hartree.

Interacting Hamiltonian: We chose as our interacting Hamiltonian the Dyall Hamiltonian, [84], the zeroth-order Hamiltonian of the n-electron valence state perturbation theory (NEVPT) [85]. To generate different interacting Hamiltonians with active space sizes $n_{\text{act}} \leq 12$, we further divided the CAS(14e, 12o) model into inactive, "inner active", and virtual spaces, with indices $\{i, j, k, l\}$, $\{u, v, w, x\}$, and $\{a, b, c, d\}$, respectively. We always included the two S $3p$ orbitals in the inner active space, and added the Fe $3d$ orbitals around the highest occupied molecular orbital to generate larger inner active spaces.

The Dyall Hamiltonian uses effective Fock operators in the inactive and virtual spaces, and

the full two-body Hamiltonian in the (inner) active space.

$$
\begin{aligned}
H_{\mathrm{Dyall}} &= \sum_{ij} f_{ij}\hat{E}_{ij} + \sum_{ab} f_{ab}\hat{E}_{ab} \\
&+ \sum_{uv} h^{\mathrm{eff}}_{uv}\hat{E}_{uv} + \frac{1}{2}\sum_{uvwx}(uv|wx)(\hat{E}_{uv}\hat{E}_{wx} - \delta_{vw}\hat{E}_{ux}) + K_{\mathrm{C,Dyall}},
\end{aligned} \tag{10}
$$

with

$$
h^{\mathrm{eff}}_{pq} = h^{\mathrm{C}}_{pq} + \sum_{i}(2(pq|ii) - (pi|iq)), \tag{11}
$$

$$
f_{pq} = h^{\mathrm{eff}}_{pq} + \sum_{uv} D_{uv}((pq|uv) - 0.5(pu|vq)), \tag{12}
$$

$$
K_{\mathrm{C,Dyall}} = K_{\mathrm{C}} + 2\sum_{i} h^{\mathrm{C}}_{ii} + \sum_{ij}(2(ii|jj) - (ij|ji)) - 2\sum_{i} f_{ii}. \tag{13}
$$

Here, h^{C}_{pq} is the effective one-electron integral including the interactions with the core orbitals defined in Supplementary Equation (7) and D_{uv} is the spin-traced one-particle density matrix in the (inner) active space. We obtained this density matrix by diagonalizing the (inner) active space problem first.

Supplementary Note 6 Local coupled cluster calculations

The coupled-cluster ansatz obtains the exact state $|\Psi_0\rangle$ from a trial state $|\Phi_0\rangle$ by the action of a wave operator encoded as an exponential of a Fock-space *cluster* operator $\hat{T}$:

$$
|\Psi_0\rangle = \exp(\hat{T})\,|\Phi_0\rangle\,; \tag{14}
$$

in practice $|\Phi_0\rangle$ is almost always a single (Hartree-Fock) determinant. The cluster operator is usually written as a sum of (particle-hole) excitations $\hat{T} = \hat{T}_1 + \hat{T}_2 + \hat{T}_3 \ldots$, where $\hat{T}_1$ creates single excitations, $\hat{T}_2$ creates double excitations, etc. Note that for finite truncations of this sum, the exponential still ensures that excitations of large numbers of particles and holes are created, and one can have Ψ_0 such that as $L \to \infty$, $\langle\Phi_0|\Psi_0\rangle = 0$. Ψ_0 which can be accurately represented by (low-order) truncations of $\hat{T}$ are typically called single-reference problems.

For quantitative accuracy in such single-reference chemical systems, empirical studies show that $\hat{T}$ must include at least 1- and 2-body excitation operators ("coupled-cluster singles and doubles", CCSD) augmented by an approximate treatment of 3-body excitations, such as in the CCSD(T) method[86, 87]. Higher order excitations, including quadruples, quintuples, etc., can also be included for small molecules, as shown by the data in the main text [88].

The local CCSD(T) method (DLPNO-CCSD(T)) illustrated in the main text [89, 90] defines the cluster operator excitations from localized orbitals occupied in $|\Phi_0\rangle$ to orbital- and orbital-pair-specific unoccupied orbitals; this allows to encode the cluster operator with $O(L^2)$ terms. Further pruning of the $\hat{T}$ operator to short-range 2- and 3-body clusters results in the linear size complexity of the cluster operator and linear complexity of its solver. To reduce the discretization error, the cluster operator is augmented by explicitly correlated[4] 2-body excitations[90].

6.1 The coupled-cluster-based protocol for the enthalpy of formation of n-alkanes

The explicitly correlated local CCSD(T) (DLPNO-CCSD(T)-F12[90]) method was used to compute the standard enthalpies of formation of n-alkanes at 298.15 K largely following the procedure benchmarked in Ref. 91. The optimal geometries for n-alkanes were determined at the Kohn-Sham DFT (B3LYP/pc-2) level of theory. The electronic atomization energy was computed as

$$\Delta E_{\text{CC}}(\text{C}_m\text{H}_{2m+2}) = mE_{\text{CC}}(\text{C}) + (2m+2)E_{\text{HF}}(\text{H}) - E_{\text{CC}}(\text{C}_m\text{H}_{m+2}), \tag{15}$$

where E_{HF} and E_{CC} denote the Hartree-Fock/cc-pVTZ-F12 and the coupled-cluster electronic energies, respectively. The latter was evaluated as

$$E_{\text{CC}} = E_{\text{DLPNO-CCSD(T)-F12(fc)/cc-pVTZ-F12}} + (E_{\text{DLPNO-CCSD(T)(ae)/cc-pCVTZ}} - E_{\text{DLPNO-CCSD(T)(fc)/cc-pCVTZ}}) \tag{16}$$

where "fc"/"ae" denote the computations with the cluster operator excluding/including the core (1s) orbitals of the C atoms, respectively; the separate treatment of the core correlation effects is due to the inadequate treatment of such effects by the current explicitly correlated F12 formalism. The coupled-cluster standard enthalpy of formation at 298.15 K was then estimated as

$$\begin{aligned}\Delta_f H^{\ominus}_{\text{CC}}(\mathrm{C}_m\mathrm{H}_{2m+2}; 298.15\ \text{K}) =& \Delta E_{\text{CC}}(\mathrm{C}_m\mathrm{H}_{2m+2}) - \Delta E_{\text{ZPE}}(\mathrm{C}_m\mathrm{H}_{2m+2}) \\ &+ m\left(\Delta_f H^{\ominus}_{\text{exp}}(\mathrm{C}; 0\ \text{K}) + \Delta H^{\ominus}_{\text{exp}}(\mathrm{C\,(cr)}; 0\ \text{K} \rightarrow 298.15\ \text{K})\right) \\ &+ (2m+2)\left(\Delta_f H^{\ominus}_{\text{exp}}(\mathrm{H}; 0\ \text{K}) + \Delta H^{\ominus}_{\text{exp}}(\mathrm{H}_2; 0\ \text{K} \rightarrow 298.15\ \text{K})\right) \\ &+ \Delta H^{\ominus}_{\text{B3LYP/pc-2}}(\mathrm{C}_m\mathrm{H}_{2m+2}; 0\ \text{K} \rightarrow 298.15\ \text{K}), \end{aligned} \tag{17}$$

where $\Delta E_{\text{ZPE}}(\mathrm{C}_m\mathrm{H}_{2m+2})$ is the zero-point correction to the atomization energy obtained from the B3LYP/pc-2 harmonic vibrational frequencies scaled by 0.985[91]. $\Delta_f H^{\ominus}_{\text{exp}}(\mathrm{X}; 0\ \text{K})$ is the standard enthalpy of formation at 0 K (the experimentally-derived ACtC[92] (version 1.122r) values for carbon (X=C) and hydrogen (H) atoms are 170.028 and 51.633 kcal/mol, respectively), $\Delta H^{\ominus}_{\text{exp}}(\mathrm{X}; 0\ \text{K} \rightarrow 298.15\ \text{K})$ is the experimental standard enthalpy difference between 298.15 K and 0 K (the CODATA values for graphite (X=C(cr)) and molecular hydrogen (X=H_2) are 0.251 and 2.024 kcal/mol, respectively[93]), and
$\Delta H^{\ominus}_{\text{B3LYP/pc-2}}(\mathrm{C}_m\mathrm{H}_{2m+2}; 0\ \text{K} \rightarrow 298.15\ \text{K})$ is the standard enthalpy difference between 298.15 K and 0 K for the n-alkanes estimated at the B3LYP/pc-2 level of theory using the ideal-gas/rigid-rotor/harmonic-oscillator molecular partition functions. Experimental enthalpies of formation at 298.15 K were taken from Ref. 94.

The remaining errors of the coupled-cluster enthalpies of formation are due to primarily the residual basis set incompleteness errors, the neglect of the accounting for multiple minima on the potential energy surface, the approximate (scaled harmonic) treatment of zero-point energy, the lack of higher-order (post-CCSD(T)) correlation effects, and the neglect of the relativistic

effects and post-Born-Oppenheimer effects.

Supplementary Table 4: Errors ($\bar{\epsilon}$ in kcal/mol) of computed gas-phase enthalpy of formation for n-alkanes (C_mH_{2m+2} with $m = [2 \ldots 20]$) per carbon atom in the standard state using the DLPNO-CCSD(T)-F12 method compared to the experimental results.

m	2	4	6	8	10	12	14	16	18	20
$\bar{\epsilon}$	0.881	0.785	0.658	0.621	0.604	0.590	0.579	0.593	0.531	0.434

6.2 Computational details of the local coupled-cluster computations

All numerical experiments utilizing the DLPNO-CCSD(T)-F12 model of computation were performed using ORCA 4.2[95] on multi-core nodes with dual E5-2683v4 2.1 GHz (Broadwell) processors with a total memory of 512 GB. We demonstrated the near-linear complexity of the DLPNO-CCSD(T)-F12 algorithm by plotting the computational wall timings vs. the system size for n-alkanes with model quasi-linear geometries, ranging from $C_{20}H_{42}$ to $C_{120}H_{242}$. The following basis-set triplet (orbital, density fitting, CABS) was used: def2-TZVP, def2-TZVP/C and cc-pVDZ-F12/OptRI. The exchange operator was approximated using the chain of spheres algorithm (COSX) while the Coulomb operator was constructed using the density-fitting procedure from the def2/J basis. All these calculations utilized the `VeryTightSCF` and `TightPNO` input settings of ORCA for higher accuracies. The effective scaling exponent using the C_{100} and C_{120} data points comes out to be around 1.38. The deviation from linearity can be attributed to procedures like the construction of the density-fitted Coulomb operator, which scales at best quadratically with the system size. The near-linear complexity of the DLPNO-CCSD(T)-F12 method has now enabled accurate calculations of the total energies of systems as large as proteins. In this work, we report one such calculation on a model fragment of the nonheme iron-bicarbonate complex in photosystem II (PSII)[96, 97] that plays a key role in photosynthesis. We used the basis-set triplet of {def2-SVP, def2-SVP/RI, `uncontracted` def2-TZVPP} for this simulation with `VeryTightSCF` and `NormalPNO` input settings. Using four computer

cores and an intra-node MPI parallel programming model, the total computational time for this energy calculation involving 565 atoms, 2170 electrons and 5420 basis functions, was less than 3 days (70 hours).

Supplementary Table 5: Computational time (t in second) of the DLPNO-CCSD(T)-F12 calculations for n-alkanes (C_mH_{2m+2} with $m = [20 \ldots 120]$).

m	20	40	60	80	100	120
t	2364	6482	11292	16877	24126	31025

Supplementary Note 7 Tensor network calculations

7.1 PEPS-DMRG Hubbard calculations

The two-dimensional Hubbard model tensor network calculations were performed with `quimb` [98] using a regular rectangular PEPS [99] ansatz with open boundaries for the 4×4 lattice at half-filling and the 4×4, 4×8, and 4×16 lattices at 1/8 doping with $U = 8$ and open boundary conditions in both directions. $U(1)$ symmetry was imposed to constrain the number of electrons. Starting from a product state, an initial guess was generated using imaginary time evolution of the PEPS via a Simple Update [100] (SU) scheme. Each iteration of the SU approach comprised a sweep over all of the Trotterized terms in the Hamiltonian, iteratively applying the imaginary time evolution gate to the corresponding nearest-neighboring PEPS site tensors. To ensure convergence, many SU sweeps were performed, with $N_{sweep} \in \{100, 200, 200, 400\}$, for decreasing imaginary time evolution step sizes of $\Delta\tau \in \{0.5, 0.1, 0.05, 0.01\}$.

To further improve upon the ground state, a PEPS-DMRG ground state optimization [101, 102] was used, which mimics the variational minimization done by the MPS-DMRG algorithm by sweeping over all lattice sites and solving localized eigenproblems at each site. To set up the local optimizations, each term in the Hamiltonian is projected into a local basis by performing an approximate boundary contraction [103] of the tensor network with Hamiltonian

operators applied to the corresponding local tensors. Reuse of intermediates is used to limit the computational cost scaling for the optimization to $\mathcal{O}\left(L_x^2 L_y^2\right)$. The accuracy of the projected Hamiltonian, and thus the optimization, is controlled by the boundary bond dimension χ, which was set to $\chi = 2D^2$ for all calculations here. This choice makes the total computational cost for given optimization sweep explicitly poly(D). The contraction error for finite χ is assessed for different square clusters up to size 16×16 in Supplementary Figure 12, which shows that the contraction error is insensitive to the lattice size in this ground-state. Because of the approximate nature of the projected Hamiltonian, an exact solution of the localized eigenproblem can lead to convergence to local minima or numerical instabilities. To avoid this, only a few iterations of a iterative eigensolver are performed at each site during a sweep; alternatively, one could use a linear combination of the initial guess and exact solution of the localized eigenstates to take small steps in the direction of the global solution. The PEPS results are summarized in Supplementary Table 6.

To benchmark the 2D tensor network calculations, we performed spin-adapted MPS-based DMRG using BLOCK2 [104]. The exact energy per site and the error are estimated using the same extrapolation method as described in Supplementary Note 4.3. The MPS results are shown in Supplementary Table 7.

7.2 PEPS-VMC Hubbard calculations

In addition to the PEPS-DMRG calculations, we also used the variational Monte Carlo method (VMC) to obtain PEPS energies at bond dimensions ≥ 5 for the 16×4 model [105, 106, 107]. This method will be denoted PEPS-VMC. In this approach, the PEPS wavefunction is sampled by Markov Chain Monte Carlo, and the weight of a configuration is computed by contracting a single-layer PEPS tensor network; the accuracy of the contraction is controlled by a boundary bond dimension χ. Both physical observables and energy gradients are evaluated using the

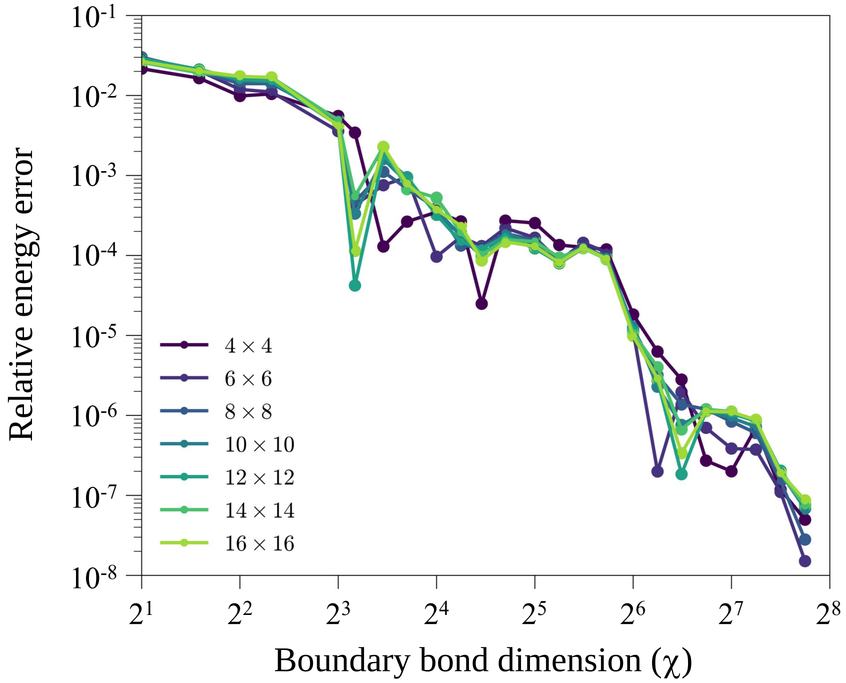

Supplementary Figure 12: Measuring the contraction error in the PEPS contraction. Relative energy error of the ground-state of the two-dimensional Hubbard model ($t = 1$ and $U = 8$) at $1/8$ doping as a function of the boundary bond dimension (χ) for different lattice sizes (L) from 4^2 to 16^2. We optimized the energies by the SU scheme with a fixed PEPS bond dimension (D) of 8. We used the energies with the largest boundary bond dimension ($\chi = 2^8$) as the reference for the error calculation. We see that the contraction error is insensitive to lattice size in this ground-state.

Supplementary Table 6: PEPS-SU, PEPS-DMRG, and PEPS-VMC energies per site of the ground state (units of t) for the two-dimensional Hubbard models with $t = 1$ and $U = 8$. D represents the maximal virtual bond dimension in the PEPS ansatz. Values in parenthesis denote MC errors at the last digit.

D	4×4, half-filling		4×4, $1/8$ doped		4×8, $1/8$ doped		4×16, $1/8$ doped		
	SU	DMRG	SU	DMRG	SU	DMRG	SU	DMRG	VMC
3			-0.5256	-0.5393	-0.5543	-0.5727	-0.5704	-0.5922	
4	-0.4054	-0.4073	-0.5576	-0.5847	-0.5865	-0.6095	-0.5962	-0.6182	-0.6182(2)
5	-0.4105	-0.4146	-0.5694	-0.6013	-0.5986	-0.6233	-0.6091	-0.6414	
6	-0.4154	-0.4189	-0.5838	-0.6089	-0.6171	-0.6370	-0.6313	-0.6526	-0.6526(1)
7	-0.4177	-0.4230							
8	-0.4180	-0.4238	-0.5979	-0.6181	-0.6285	-0.6532	-0.6415	-0.6624	-0.6626(2)
10	-0.4208	-0.4246	-0.6056	-0.6220					-0.6688(1)
12									-0.6727(1)
14									-0.6760(1)

Supplementary Table 7: MPS-DMRG energies per site of the ground state (units of t) for the two-dimensional Hubbard models with $t = 1$ and $U = 8$. D represents the maximal bond dimension in the spin-adapted MPS ansatz.

D	Discarded weight	Energy per site	D	Discarded weight	Energy per site
	4×4 at half-filling			4×4 at $1/8$ doped	
500	3.59×10^{-6}	-0.42551504	500	7.30×10^{-5}	-0.63241788
1000	2.61×10^{-7}	-0.42552519	1000	5.40×10^{-6}	-0.63260460
1500	5.80×10^{-8}	-0.42552583	1500	1.01×10^{-6}	-0.63261660
2000	1.50×10^{-8}	-0.42552589	2000	2.50×10^{-7}	-0.63261805
2500	4.12×10^{-9}	-0.42552590	2500	7.55×10^{-8}	-0.63261830
∞		-0.42552596(1)	∞		-0.6326190(1)
	4×8 at $1/8$ doped			4×16 at $1/8$ doped	
500	3.66×10^{-4}	-0.66601570	6000	2.27×10^{-5}	-0.68515566
1000	1.87×10^{-4}	-0.66842805	7000	1.87×10^{-5}	-0.68522094
1500	1.13×10^{-4}	-0.66918879	8000	1.62×10^{-5}	-0.68526802
2000	7.59×10^{-5}	-0.66952323	9000	1.41×10^{-5}	-0.68530352
2500	5.38×10^{-5}	-0.66969776	∞		-0.68555(5)
∞		-0.67047(15)			

importance sampling. In the optimization process of PEPS-VMC, the initial guess is from the aforementioned SU approach, and then the state is further optimized by stochastic gradient optimization. The optimization step length δ is gradually reduced according to the magnitude of the energy change, from $\delta = 0.005$ to 0.0005. The optimization is terminated when the energy does not improve (converged up to 3 decimal places) after 50 optimization steps with $\delta = 0.0005$. The cost of each optimization iteration is $O(L_x^2 L_y^2 \chi^2 D^4)$, and we find $\chi = 3D$ works well to produce converged results for all calculations given in Supplementary Table 6.

The PEPS-VMC method gives consistent results with the PEPS-DMRG method within the statistical error of Monte Carlo sampling. The orange curve shown in the bottom panel of Fig. 4B in the main text (16×4 Hubbard model) denotes PEPS-DMRG energies for $D < 5$ and PEPS-VMC energies for $D \geq 5$.

7.3 3D Heisenberg model PEPS reference calculations

The 3D anti-ferromagnetic Heisenberg model tensor network simulations were performed with `quimb` [98], using a PEPS [99] ansatz on a cubic lattice with open boundary conditions and Simple Update [100] (SU) style imaginary time evolution. Although more accurate energies could be obtained by variational minimization, we have not performed those calculations in this work due to the large number of tensors to optimize (up to 1000). The energies of the resulting states are calculated using a basic boundary contraction scheme.

For each cube length $L{=}\{3, 4, 6, 8, 10\}$, with total number of sites $N{=}L{\times}L{\times}L$, separate ansatz states with virtual bond dimensions $D{=}2, 3, 4$ were generated. The SU scheme consists of a fixed schedule of 5 sweeps at each imaginary time step $\tau = 10^p$ for $p = \{1.0, 0.9, 0.8, \ldots -3.0\}$ starting from a random PEPS regardless of D. Each sweep uses a randomly generated edge coloring of the underlying graph to Trotterize the gates.

The boundary contraction algorithm contracts each face of the cube inwards as a 2D PEPS,

eagerly compressing between the tensors as they are contracted into the next plane. No gauging is performed other than performing the SVDs in the reduced basis and ensuring the truncated singular values are absorbed evenly. The actual error values and computational costs are taken using $\chi = \{8, 14, 20\}$ for $D = \{2, 3, 4\}$, respectively, corresponding to $\chi = D^2 + 4$ rounded to the nearest even integer. Note that for SU the cost of computing the energy (or other local quantities) completely dominates the cost of actually performing the optimization. The uncertainty on the error itself is estimated using the standard deviation of energies computed with $\chi = \{10, 12, ..., 20\}$.

The reference energies were computed with ALPS [108], using stochastic series expansion quantum Monte Carlo [109, 110, 111], and were converged to an accuracy of $\approx 10^{-5}$ using a temperature of $T = 0.003J$, where J is the coupling strength.

Supplementary References

[1] Lee, S., Lee, J. & Chan, G. K.-L. Collection of papers referring exponential quantum advantage in quantum chemistry. `https://github.com/seunghoonlee89/Refs_EQA_GSQC` (2023).

[2] Gharibian, S. & Gall, F. L. Dequantizing the quantum singular value transformation: Hardness and applications to quantum chemistry and the quantum pcp conjecture. *arXiv preprint arXiv:2111.09079* (2021).

[3] Cade, C., Folkertsma, M. & Weggemans, J. Complexity of the guided local hamiltonian problem: Improved parameters and extension to excited states. *arXiv preprint arXiv:2207.10097* (2022).

[4] Kong, L., Bischoff, F. A. & Valeev, E. F. Explicitly correlated R12/F12 methods for electronic structure. *Chemical reviews* **112**, 75–107 (2012).

[5] Cramer, C. J. *Essentials of computational chemistry: theories and models* (John Wiley & Sons, 2013).

[6] Shavitt, I. & Bartlett, R. J. *Many-body methods in chemistry and physics: MBPT and coupled-cluster theory* (Cambridge university press, 2009).

[7] Motta, M. & Zhang, S. Ab initio computations of molecular systems by the auxiliary-field quantum monte carlo method. *Wiley Interdisciplinary Reviews: Computational Molecular Science* **8**, e1364 (2018).

[8] Becca, F. & Sorella, S. *Quantum Monte Carlo approaches for correlated systems* (Cambridge University Press, 2017).

[9] Chan, G. K.-L. & Sharma, S. The density matrix renormalization group in quantum chemistry. *Annual review of physical chemistry* **62**, 465–481 (2011).

[10] Orús, R. Tensor networks for complex quantum systems. *Nature Reviews Physics* **1**, 538–550 (2019).

[11] Pfau, D., Spencer, J. S., Matthews, A. G. & Foulkes, W. M. C. Ab initio solution of the many-electron schrödinger equation with deep neural networks. *Physical Review Research* **2**, 033429 (2020).

[12] Hermann, J., Schätzle, Z. & Noé, F. Deep-neural-network solution of the electronic schrödinger equation. *Nature Chemistry* **12**, 891–897 (2020).

[13] Mazziotti, D. A. Variational two-electron reduced density matrix theory for many-electron atoms and molecules: Implementation of the spin-and symmetry-adapted t 2 condition through first-order semidefinite programming. *Physical Review A* **72**, 032510 (2005).

[14] Eriksen, J. J. *et al.* The ground state electronic energy of benzene. *The journal of physical chemistry letters* **11**, 8922–8929 (2020).

[15] Williams, K. T. *et al.* Direct comparison of many-body methods for realistic electronic hamiltonians. *Physical Review X* **10**, 011041 (2020).

[16] Motta, M. *et al.* Towards the solution of the many-electron problem in real materials: Equation of state of the hydrogen chain with state-of-the-art many-body methods. *Physical Review X* **7**, 031059 (2017).

[17] Lin, L. & Tong, Y. Near-optimal ground state preparation. *Quantum* **4**, 372 (2020).

[18] Kitaev, A. Y., Shen, A., Vyalyi, M. N. & Vyalyi, M. N. *Classical and quantum computation*. 47 (American Mathematical Soc., 2002).

[19] Aharonov, D., Gottesman, D., Irani, S. & Kempe, J. The power of quantum systems on a line. *Communications in mathematical physics* **287**, 41–65 (2009).

[20] Gottesman, D. & Irani, S. The quantum and classical complexity of translationally invariant tiling and hamiltonian problems. In *2009 50th Annual IEEE Symposium on Foundations of Computer Science*, 95–104 (IEEE, 2009).

[21] Bausch, J. & Crosson, E. Analysis and limitations of modified circuit-to-Hamiltonian constructions. *Quantum* **2**, 94 (2018). URL `https://doi.org/10.22331/q-2018-09-19-94`.

[22] Hastings, M. B. An area law for one-dimensional quantum systems. *Journal of Statistical Mechanics: Theory and Experiment* **2007**, P08024–P08024 (2007). URL `https://doi.org/10.1088/1742-5468/2007/08/p08024`.

[23] Arad, I., Kitaev, A., Landau, Z. & Vazirani, U. An area law and sub-exponential algorithm for 1d systems (2013). `1301.1162`.

[24] Hastings, M. B. Entropy and entanglement in quantum ground states. *Physical Review B* **76**, 035114 (2007).

[25] Landau, Z., Vazirani, U. & Vidick, T. A polynomial time algorithm for the ground state of one-dimensional gapped local hamiltonians. *Nature Physics* **11**, 566–569 (2015).

[26] Huang, Y. A polynomial-time algorithm for the ground state of one-dimensional gapped hamiltonians (2015). `1406.6355`.

[27] Chubb, C. T. & Flammia, S. T. Computing the degenerate ground space of gapped spin chains in polynomial time. *Chicago Journal of Theoretical Computer Science* **2016** (2016).

[28] Arad, I., Landau, Z., Vazirani, U. & Vidick, T. Rigorous rg algorithms and area laws for low energy eigenstates in 1d. *Communications in Mathematical Physics* **356**, 65–105 (2017).

[29] Roberts, B., Vidick, T. & Motrunich, O. I. Implementation of rigorous renormalization group method for ground space and low-energy states of local hamiltonians. *Phys. Rev. B* **96**, 214203 (2017).

[30] Abrahamsen, N. A polynomial-time algorithm for ground states of spin trees (2020). `1907.04862`.

[31] Corboz, P. & Vidal, G. Fermionic multiscale entanglement renormalization ansatz. *Physical Review B* **80**, 165129 (2009).

[32] Evenbly, G. & Vidal, G. Scaling of entanglement entropy in the (branching) multiscale entanglement renormalization ansatz. *Physical Review B* **89**, 235113 (2014).

[33] Stojevic, V., Haegeman, J., McCulloch, I., Tagliacozzo, L. & Verstraete, F. Conformal data from finite entanglement scaling. *Physical Review B* **91**, 035120 (2015).

[34] Mortier, Q., Schuch, N., Verstraete, F. & Haegeman, J. Resolving fermi surfaces with tensor networks. *arXiv preprint arXiv:2008.11176* (2020).

[35] Albash, T. & Lidar, D. A. Adiabatic quantum computation. *Reviews of Modern Physics* **90**, 015002 (2018).

[36] Elgart, A. & Hagedorn, G. A. A note on the switching adiabatic theorem. *Journal of Mathematical Physics* **53**, 102202 (2012).

[37] Jansen, S., Ruskai, M.-B. & Seiler, R. Bounds for the adiabatic approximation with applications to quantum computation. *Journal of Mathematical Physics* **48**, 102111 (2007).

[38] Bachmann, S., De Roeck, W. & Fraas, M. Adiabatic theorem for quantum spin systems. *Physical review letters* **119**, 060201 (2017).

[39] Nielsen, M. A. & Chuang, I. *Quantum computation and quantum information* (Cambridge University Press;, 2011).

[40] Babbush, R. *et al.* Encoding electronic spectra in quantum circuits with linear T complexity. *Physical Review X* **8**, 041015 (2018).

[41] Luis, A. & Peřina, J. Optimum phase-shift estimation and the quantum description of the phase difference. *Physical review A* **54**, 4564 (1996).

[42] Sanders, Y. R. *et al.* Compilation of fault-tolerant quantum heuristics for combinatorial optimization. *PRX Quantum* **1**, 020312 (2020).

[43] Kaiser, J. & Schafer, R. On the use of the I_0-sinh window for spectrum analysis. *IEEE Transactions on Acoustics, Speech, and Signal Processing* **28**, 105–107 (1980).

[44] Haghshenas, R., Lan, W.-W., Gong, S.-S. & Sheng, D. Quantum phase diagram of spin-1 j 1- j 2 heisenberg model on the square lattice: An infinite projected entangled-pair state and density matrix renormalization group study. *Physical Review B* **97**, 184436 (2018).

[45] Liu, W.-Y. *et al.* Gapless quantum spin liquid and global phase diagram of the spin-1/2j_1-j_2 square antiferromagnetic heisenberg model. *arXiv preprint arXiv:2009.01821* (2020).

[46] Zheng, B.-X. *et al.* Stripe order in the underdoped region of the two-dimensional hubbard model. *Science* **358**, 1155–1160 (2017).

[47] Liang, X. *et al.* Solving frustrated quantum many-particle models with convolutional neural networks. *Physical Review B* **98**, 104426 (2018).

[48] Sorella, S. The phase diagram of the hubbard model by variational auxiliary field quantum monte carlo. *arXiv preprint arXiv:2101.07045* (2021).

[49] White, S. R. & Martin, R. L. Ab initio quantum chemistry using the density matrix renormalization group. *The Journal of chemical physics* **110**, 4127–4130 (1999).

[50] Nakatani, N. & Chan, G. K.-L. Efficient tree tensor network states (ttns) for quantum chemistry: Generalizations of the density matrix renormalization group algorithm. *The Journal of chemical physics* **138**, 134113 (2013).

[51] Gunst, K., Verstraete, F., Wouters, S., Legeza, O. & Van Neck, D. T3ns: three-legged tree tensor network states. *Journal of chemical theory and computation* **14**, 2026–2033 (2018).

[52] Baiardi, A. & Reiher, M. The density matrix renormalization group in chemistry and molecular physics: Recent developments and new challenges. *The Journal of Chemical Physics* **152**, 040903 (2020).

[53] Haghshenas, R., Cui, Z.-H. & Chan, G. K.-L. Numerical continuum tensor networks in two dimensions. *Physical Review Research* **3**, 023057 (2021).

[54] O'Rourke, M. J. & Chan, G. K. Entanglement in the quantum phases of an unfrustrated rydberg atom array. *arXiv preprint arXiv:2201.03189* (2022).

[55] Georges, A., Kotliar, G., Krauth, W. & Rozenberg, M. J. Dynamical mean-field theory of strongly correlated fermion systems and the limit of infinite dimensions. *Reviews of Modern Physics* **68**, 13 (1996).

[56] Knizia, G. & Chan, G. K.-L. Density matrix embedding: A simple alternative to dynamical mean-field theory. *Physical review letters* **109**, 186404 (2012).

[57] Lin, N., Marianetti, C., Millis, A. J. & Reichman, D. R. Dynamical mean-field theory for quantum chemistry. *Physical review letters* **106**, 096402 (2011).

[58] Zgid, D. & Chan, G. K.-L. Dynamical mean-field theory from a quantum chemical perspective. *The Journal of chemical physics* **134**, 094115 (2011).

[59] Cui, Z.-H., Zhu, T. & Chan, G. K.-L. Efficient implementation of ab initio quantum embedding in periodic systems: Density matrix embedding theory. *J. Chem. Theory*

Comput. **16**, 119–129 (2020). URL `https://pubs.acs.org/doi/full/10.1021/acs.jctc.9b00933`.

[60] Welborn, M., Tsuchimochi, T. & Van Voorhis, T. Bootstrap embedding: An internally consistent fragment-based method. *The Journal of Chemical Physics* **145**, 074102 (2016).

[61] Biroli, G. & Kotliar, G. Reply to "comment on 'cluster methods for strongly correlated electron systems'". *Physical Review B* **71**, 037102 (2005).

[62] Li, Z. & Chan, G. K.-L. Spin-projected matrix product states: Versatile tool for strongly correlated systems. *Journal of chemical theory and computation* **13**, 2681–2695 (2017).

[63] Li, Z., Guo, S., Sun, Q. & Chan, G. K.-L. Electronic landscape of the p-cluster of nitrogenase as revealed through many-electron quantum wavefunction simulations. *Nature chemistry* **11**, 1026–1033 (2019).

[64] Li, Z., Li, J., Dattani, N. S., Umrigar, C. & Chan, G. K.-L. The electronic complexity of the ground-state of the femo cofactor of nitrogenase as relevant to quantum simulations. *The Journal of chemical physics* **150**, 024302 (2019).

[65] Sharma, S., Sivalingam, K., Neese, F. & Chan, G. K.-L. Low-energy spectrum of iron–sulfur clusters directly from many-particle quantum mechanics. *Nature chemistry* **6**, 927–933 (2014).

[66] Peters, J. W. *et al.* Redox-dependent structural changes in the nitrogenase p-cluster. *Biochemistry* **36**, 1181–1187 (1997).

[67] Ohki, Y., Sunada, Y., Honda, M., Katada, M. & Tatsumi, K. Synthesis of the p-cluster

inorganic core of nitrogenases. *Journal of the American Chemical Society* **125**, 4052–4053 (2003).

[68] Spatzal, T. *et al.* Evidence for interstitial carbon in nitrogenase femo cofactor. *Science* **334**, 940–940 (2011).

[69] Bytautas, L., Ivanic, J. & Ruedenberg, K. Split-localized orbitals can yield stronger configuration interaction convergence than natural orbitals. *The Journal of chemical physics* **119**, 8217–8224 (2003).

[70] Pipek, J. & Mezey, P. G. A fast intrinsic localization procedure applicable for abinitio and semiempirical linear combination of atomic orbital wave functions. *The Journal of Chemical Physics* **90**, 4916–4926 (1989).

[71] Becke, A. D. Density-functional exchange-energy approximation with correct asymptotic behavior. *Physical review A* **38**, 3098 (1988).

[72] Perdew, J. P. Density-functional approximation for the correlation energy of the inhomogeneous electron gas. *Physical Review B* **33**, 8822 (1986).

[73] Weigend, F. & Ahlrichs, R. Balanced basis sets of split valence, triple zeta valence and quadruple zeta valence quality for h to rn: Design and assessment of accuracy. *Physical Chemistry Chemical Physics* **7**, 3297–3305 (2005).

[74] Liu, W. Ideas of relativistic quantum chemistry. *Molecular Physics* **108**, 1679–1706 (2010).

[75] Saue, T. Relativistic hamiltonians for chemistry: A primer. *ChemPhysChem* **12**, 3077–3094 (2011).

[76] Peng, D. & Reiher, M. Exact decoupling of the relativistic fock operator. *Theoretical Chemistry Accounts* **131**, 1–20 (2012).

[77] Becke, A. D. Becke's three parameter hybrid method using the lyp correlation functional. *J. Chem. Phys* **98**, 5648–5652 (1993).

[78] Lee, C., Yang, W. & Parr, R. G. Development of the colle-salvetti correlation-energy formula into a functional of the electron density. *Physical review B* **37**, 785 (1988).

[79] Stephens, P. J., Devlin, F. J., Chabalowski, C. F. & Frisch, M. J. Ab initio calculation of vibrational absorption and circular dichroism spectra using density functional force fields. *The Journal of physical chemistry* **98**, 11623–11627 (1994).

[80] Klamt, A. & Schüürmann, G. Cosmo: a new approach to dielectric screening in solvents with explicit expressions for the screening energy and its gradient. *Journal of the Chemical Society, Perkin Transactions 2* 799–805 (1993).

[81] Cao, L. & Ryde, U. Influence of the protein and dft method on the broken-symmetry and spin states in nitrogenase. *International Journal of Quantum Chemistry* **118**, e25627 (2018).

[82] Olivares-Amaya, R. *et al.* The ab-initio density matrix renormalization group in practice. *The Journal of chemical physics* **142**, 034102 (2015).

[83] Pople, J. A. Nobel lecture: Quantum chemical models. *Reviews of Modern Physics* **71**, 1267 (1999).

[84] Dyall, K. G. The choice of a zeroth-order hamiltonian for second-order perturbation theory with a complete active space self-consistent-field reference function. *The Journal of chemical physics* **102**, 4909–4918 (1995).

[85] Angeli, C., Cimiraglia, R., Evangelisti, S., Leininger, T. & Malrieu, J.-P. Introduction of n-electron valence states for multireference perturbation theory. *The Journal of Chemical Physics* **114**, 10252–10264 (2001).

[86] Raghavachari, K., Trucks, G. W., Pople, J. A. & Head-Gordon, M. A FIFTH-ORDER PERTURBATION COMPARISON OF ELECTRON CORRELATION THEORIES. *Chemical physics letters* **157**, 479–483 (1989).

[87] Watts, J. D., Gauss, J. & Bartlett, R. J. Coupled-cluster methods with noniterative triple excitations for restricted open-shell Hartree–Fock and other general single determinant reference functions. Energies and analytical gradients. *The Journal of Chemical Physics* **98**, 8718–8733 (1993).

[88] Chan, G. K.-L., Kállay, M. & Gauss, J. State-of-the-art density matrix renormalization group and coupled cluster theory studies of the nitrogen binding curve. *The Journal of chemical physics* **121**, 6110–6116 (2004).

[89] Riplinger, C., Pinski, P., Becker, U., Valeev, E. F. & Neese, F. Sparse maps—A systematic infrastructure for reduced-scaling electronic structure methods. II. Linear scaling domain based pair natural orbital coupled cluster theory. *The Journal of chemical physics* **144**, 024109 (2016).

[90] Kumar, A., Neese, F. & Valeev, E. F. Explicitly correlated coupled cluster method for accurate treatment of open-shell molecules with hundreds of atoms. *The Journal of chemical physics* **153**, 094105 (2020).

[91] Karton, A., Gruzman, D. & Martin, J. M. L. Benchmark Thermochemistry of the $C_n H_{2n+2}$ Alkane Isomers (n = 2-8) and Performance of DFT and Composite Ab Initio

Methods for Dispersion-Driven Isomeric Equilibria. *The Journal of Physical Chemistry A* **113**, 8434–8447 (2009).

[92] Ruscic, B. *et al.* Active Thermochemical Tables: Thermochemistry for the 21st century. *Journal of Physics: Conference Series* **16**, 561–570 (2005).

[93] Cox, J. D., Wagman, D. D. & Medvedev, V. A. V. A. *CODATA Key Values for Thermodynamics*. CODATA Series on Thermodynamic Properties. (Hemisphere Pub. Corp., New York, 1989).

[94] Prosen, E. & Rossini, F. Heats of combustion and formation of the paraffin hydrocarbons at 25 degrees C. *Journal of Research of the National Bureau of Standards* **34**, 263 (1945).

[95] Neese, F., Wennmohs, F., Becker, U. & Riplinger, C. The orca quantum chemistry program package. *The Journal of Chemical Physics* **152**, 224108 (2020). URL https://doi.org/10.1063/5.0004608. https://doi.org/10.1063/5.0004608.

[96] Müh, F. & Zouni, A. The nonheme iron in photosystem II. *Photosynthesis Research* **116**, 295–314 (2013).

[97] Saitow, M., Becker, U., Riplinger, C., Valeev, E. F. & Neese, F. A new near-linear scaling, efficient and accurate, open-shell domain-based local pair natural orbital coupled cluster singles and doubles theory. *The Journal of chemical physics* **146**, 164105 (2017). URL http://aip.scitation.org/doi/10.1063/1.4981521.

[98] Gray, J. quimb: A python package for quantum information and many-body calculations. *Journal of Open Source Software* **3**, 819 (2018).

[99] Verstraete, F. & Cirac, J. I. Renormalization algorithms for quantum-many body systems in two and higher dimensions. *arXiv preprint cond-mat/0407066* (2004).

[100] Jiang, H.-C., Weng, Z.-Y. & Xiang, T. Accurate determination of tensor network state of quantum lattice models in two dimensions. *Physical review letters* **101**, 090603 (2008).

[101] Verstraete, F., Murg, V. & Cirac, J. I. Matrix product states, projected entangled pair states, and variational renormalization group methods for quantum spin systems. *Advances in physics* **57**, 143–224 (2008).

[102] Hyatt, K. & Stoudenmire, E. M. Dmrg approach to optimizing two-dimensional tensor networks. *arXiv preprint arXiv:1908.08833* (2019).

[103] Lubasch, M., Cirac, J. I. & Banuls, M.-C. Unifying projected entangled pair state contractions. *New Journal of Physics* **16**, 033014 (2014).

[104] Zhai, H. & Chan, G. K.-L. Low communication high performance ab initio density matrix renormalization group algorithms. *J. Chem. Phys* **154**, 224116 (2021).

[105] Liu, W.-Y., Dong, S.-J., Han, Y.-J., Guo, G.-C. & He, L. Gradient optimization of finite projected entangled pair states. *Physical Review B* **95**, 195154 (2017).

[106] Liu, W.-Y., Huang, Y.-Z., Gong, S.-S. & Gu, Z.-C. Accurate simulation for finite projected entangled pair states in two dimensions. *Physical Review B* **103**, 235155 (2021).

[107] Liu, W.-Y. *et al.* Gapless quantum spin liquid and global phase diagram of the spin-1/2 j1-j2 square antiferromagnetic heisenberg model. *Science Bulletin* (2022).

[108] Bauer, B. *et al.* The alps project release 2.0: open source software for strongly correlated systems. *Journal of Statistical Mechanics: Theory and Experiment* **2011**, P05001 (2011).

[109] Sandvik, A. W. & Kurkijärvi, J. Quantum monte carlo simulation method for spin systems. *Physical Review B* **43**, 5950 (1991).

[110] Evertz, H. G., Lana, G. & Marcu, M. Cluster algorithm for vertex models. *Physical review letters* **70**, 875 (1993).

[111] Sandvik, A. W. Finite-size scaling of the ground-state parameters of the two-dimensional heisenberg model. *Physical Review B* **56**, 11678 (1997).
